# Supplementary material for: Physiologically Based Pharmacokinetic Modelling of Serum 25-Hydroxyvitamin D Concentrations in Schoolchildren Receiving Weekly Oral Vitamin D3 Supplementation
Source: Nutrients. 2025 Sep 23;17(19):3028. doi: 10.3390/nu17193028 (PMC12525942; doi:10.3390/nu17193028)
Supplement: Supplementary file 1 [file nutrients-17-03028-s001.zip › nutrients-3829772-supplementary.pdf]

Physiologically-based Pharmacokinetic Modelling of Serum 25-hydroxyvitamin D concentrations in Schoolchildren Receiving Weekly Oral Vitamin D Supplementation

Nadda Muhamad[1,2,3], Neil Walker[4], Keren Middelkoop[5,6], Davaasambuu Ganmaa[7], Adrian R Martineau[4], Tao You[1,2,8,*]

[1] Department of Pharmacology and Therapeutics, University of Liverpool, Liverpool, L69 7BE, UK

[2] Centre of Excellence in Long-acting Therapeutics (CELT), University of Liverpool, Liverpool, L69 7BE, UK

[3] Department of Biomedicine and Health Informatics, Faculty of Pharmacy, Silpakorn University, Nakhon Pathom 73000, Thailand

[4] Blizard Institute, Faculty of Medicine and Dentistry, Queen Mary University of London, London E1 2AT, UK

[5] Institute of Infectious Disease and Molecular Medicine, University of Cape Town, Cape Town, South Africa

[6] Desmond Tutu HIV Centre, Department of Medicine, University of Cape Town, Cape Town, South Africa

[7] Department of Nutrition, Harvard School of Public Health, Boston, MA, USA

[8] Beyond Consulting Ltd. 14 Tytherington Park Road, Macclesfield, Cheshire, SK10 2EL, UK

* Correspondence: tao.you@liverpool.ac.uk

Supplementary Materials

Figure S1. Flowchart of Mongolian children selection for model simulations.

Figure S2. Model diagram for models 1-8 and 11.

Figure S3. Model diagram for models 9 and 10.

Figure S4. Serum 25(OH)D concentrations after repeated weekly dosing of 250 µg vitamin D_3.

Figure S5. Baseline serum 25(OH)D levels vs. BMI-for-age Z-score for 77 children in the treatment group.

Figure S6. The scatter plot for pharmacokinetic parameters for the final model and various reported covariates.

Figure S7. Objective function value v Kp25lm. The objective function is fitted at different Kp25lm values: 1, 2, 3, 4, 6, 8.

Figure S8. The fitted 77 children.

Figure S9. Simulation of serum 25(OH)D concentrations in 454 Cape Town children in the treatment group.

Figure S10. The distribution of residual error at 3 years: observation – population prediction at the 50th percentile.

Figure S11. The distribution of the range (i.e. 97.5^{th} – 2.5^{th} percentiles) at 1, 2, and 3 years.

Figure S12. The scatter plots between the 2.5th – 97.5th percentiles range and baseline bodyweight and ZBMI.

Figure S13. Comparison of the treated Mongolian children with treated Cape Town children used to fit the model.

Figure S14. Simulation of serum 25(OH)D concentrations in Mongolian children receiving a weekly dose of 350 µg vitamin D_3.

Figure S15. Serum 25(OH)D in the control group of the ViDiKids safety study (n=105) changes with time.

Figure S16: Simulation of serum 25(OH)D concentration at 3 years with weekly 250 µg vitamin D_3 dosing at varying values of CL_{max} and C_{50}.

Figure S17. 25(OH)D clearance rate $\frac{CL_{max} \times C_{25D}{}^{\gamma}}{C_{50}{}^{\gamma} + C_{25D}{}^{\gamma}} \times C_{25D}$ vs 25(OH)D.

Table S1. Baseline characteristics of the study population.

Table S2. Physiological parameters used for paediatric compartmental lumping.

Table S3. Physiological parameters of the PBPK model for paediatrics after compartments are lumped together.

Table S4. Drug-specific parameters for model development.

Table S5. Model development for 25(OH)D paediatric pharmacokinetics.

Table S6. 25(OH)D model fitting results.

Supplementary Figures

Figure S1. Flowchart of Mongolian children selection for model simulations. Schoolchildren aged 6 to 13 years from participating schools in Ulaanbaatar, Mongolia, were recruited from September 2015 to March 2017 to the randomised controlled trials of vitamin D supplementation to prevent tuberculosis infection. Over the 3-year study period, each child randomised to the intervention arm received a weekly oral capsule containing 350μg (14,000 IU) vitamin D_3 or a placebo. Blood samples were collected at baseline and at the end of the third year. Serum 25(OH)D_3 concentrations were determined using an enzyme-linked fluorescent assay [16]. The participants in the treatment group with 25(OH)D concentrations above the lower limit of quantification (14.2 nmol/L) at both baseline and follow-up were included in the model simulation using the final model

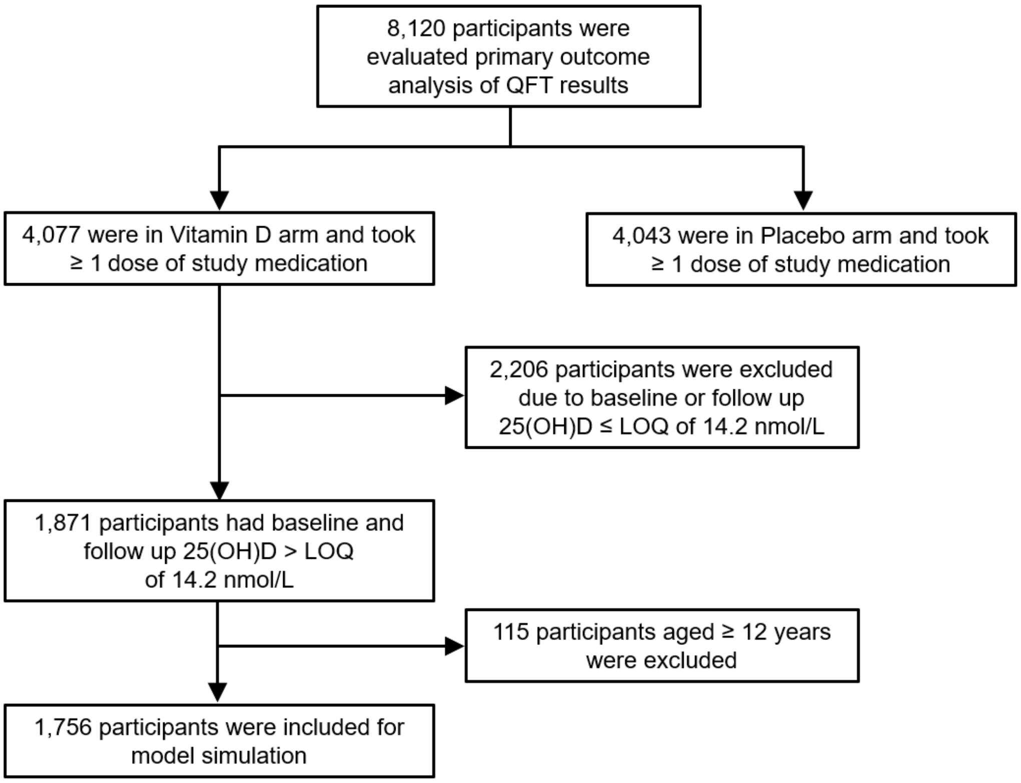

Figure S2. Model diagram for models 1-8 and 11, adapted from our previous publication [13]. The model considers 5 compartments for vitamin D including venous blood, arterial blood, liver, and the rest of body (i.e. all non-eliminating organs) and 4 compartments for 25(OH)D, including venous blood, arterial blood, liver, and the rest of body (i.e. all non-eliminating organs). Vitamin D_3 supplement enters the body via gastrointestinal tract (GI) and passes through the liver to either enter the venous blood or to be metabolised into 25(OH)D_3. Endogenously synthesised vitamin D_3 is assumed to enter the liver at a constant rate ENDOG, which varies for each person. The equation of 25(OH)D_3 clearance (CL_R) is listed in the bottom right corner. GI: gastrointestinal tract. Q_{co}: Cardiac output (L/h). Q_{rb}: blood flow into and out of the rest of body. Q_l: blood flow into and out of the liver. Kp_{rb} and Kp_{25rb}: distribution coefficient between venous blood and the rest of body for vitamin D_3 and 25(OH)D_3, respectively. Kp_l and Kp_{25l}: distribution coefficient between venous blood and the liver for vitamin D_3 and 25(OH)D_3, respectively. F_m: fraction of vitamin D_3 metabolised into 25(OH)D_3. CL_H: hepatic clearance rate (L/h). C_{VD}: concentration of hepatic vitamin D_3.

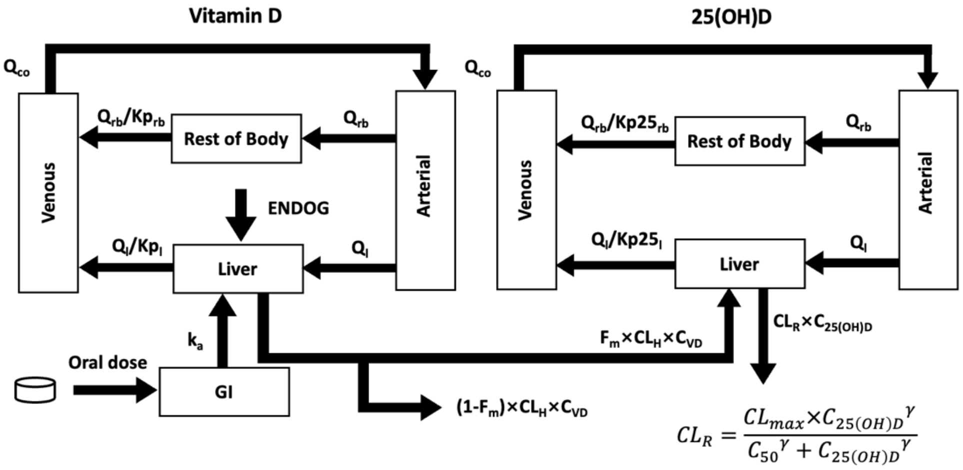

$$CL_R = \frac{CL_{max} \times C_{25(OH)D}{}^{\gamma}}{C_{50}{}^{\gamma} + C_{25(OH)D}{}^{\gamma}}$$

$$ENDOG = \frac{CL_{max} \times D25_{BASE}{}^{\gamma}}{C_{50}{}^{\gamma} + D25_{BASE}{}^{\gamma}} \times D25_{BASE} \times 3$$

$$\frac{dA_{dep}}{dt} = -k_a \times A_{dep}$$

$$\frac{dA_{ven}}{dt} = \frac{Q_l \times A_l}{V_l} \times \frac{1}{K_{pl}} + \frac{Q_{rb} \times A_{rb}}{V_{rb}} \times \frac{1}{K_{prb}} - \frac{Q_{co} \times A_{ven}}{V_{ven}}$$

$$\frac{dA_l}{dt} = -\frac{Q_l \times A_l}{V_l} \times \frac{1}{K_{pl}} + \frac{Q_l \times A_{art}}{V_{art}} - \frac{MCL_H \times A_l}{V_l} \times \frac{1}{K_{pl}} + ENDOG + k_a \times A_{dep}$$

$$\frac{dA25_{ven}}{dt} = \frac{Q_l \times A25_l}{V_l} \times \frac{1}{K_{p25l}} + \frac{Q_{rb} \times A25_{rb}}{V_{rb}} \times \frac{1}{K_{p25rb}} - \frac{Q_{co} \times A25_{ven}}{V_{ven}}$$

$$\frac{dA25_l}{dt} = -\frac{Q_l \times A25_l}{V_l} \times \frac{1}{K_{p25l}} + \frac{Q_l \times A25_{art}}{V_{art}} + \frac{MCL_H \times A_l}{V_l} \times \frac{1}{K_{pl}} \times \frac{1}{3} - \frac{CL_{max} \times \left(\frac{A25_l}{V_l} \times \frac{1}{K_{p25l}}\right)^{\gamma}}{C_{50}{}^{\gamma} + \left(\frac{A25_l}{V_l} \times \frac{1}{K_{p25l}}\right)^{\gamma}} \times \frac{A25_l}{V_l} \times \frac{1}{K_{p25l}}$$

$$\frac{dA25_{rb}}{dt} = \frac{Q_{rb} \times A25_{art}}{V_{art}} - \frac{Q_{rb} \times A25_{rb}}{V_{rb}} \times \frac{1}{K_{p25rb}}$$

$$\frac{dA25_{art}}{dt} = \frac{Q_{co} \times A25_{ven}}{V_{ven}} - \frac{Q_l \times A25_{art}}{V_{art}} - \frac{Q_{rb} \times A25_{art}}{V_{art}}$$

Initial conditions:

$$A_{art} = \frac{\frac{Q_{co} \times A_{ven}}{V_{ven}}}{\frac{Q_l + Q_{rb}}{V_{art}}}$$

$$A_{rb} = \frac{A_{art} \times K_{prb} \times V_{rb}}{V_{art}} = \frac{\frac{\frac{Q_{co} \times A_{ven}}{V_{ven}}}{\frac{Q_l + Q_{rb}}{V_{art}}} \times K_{prb} \times V_{rb}}{V_{art}}$$

$$A_{dep}(0) = 0$$

$$A_{ven}(0) = ENDOG \times \frac{V_{ven}}{MCL_H}$$

$$A_l(0) = ENDOG \times \frac{V_l}{MCL_H} \times K_{pl}$$

$$A25_{ven}(0) = D25_{BASE} \times V_{ven}$$

$$A25_l(0) = D25_{BASE} \times V_l \times K_{p25l}$$

$$A25_{rb}(0) = D25_{BASE} \times V_{rb} \times K_{p25rb}$$

$$A25_{art}(0) = D25_{BASE} \times V_{art}$$

Figure S3. Model diagram for models 9-10, adapted from our previous publication [13]. The model considers 5 compartments for vitamin D (same as models 1-8 and 11) and 5 compartments 25(OH)D, including the arterial blood, venous blood, liver, fat mass and lean mass. Vitamin D_3 supplement enters the body via gastrointestinal tract (GI) and passes through the liver to either enter the venous blood or to be metabolised into 25(OH)D_3. Endogenously synthesised vitamin D_3 is assumed to enter the liver at a constant rate ENDOG, which varies for each person. The equation of 25(OH)D_3 clearance (CL_R) is listed in the bottom right corner. GI: gastrointestinal tract. Q_{co}: Cardiac output (L/h). Q_{rb}: blood flow into and out of the rest of body. Q_l: blood flow into and out of the liver. Q_{fm}: blood flow into and out of the fat mass. Q_{lm}: blood flow into and out of the lean mass. Kp_{rb} and Kp_{25rb}: distribution coefficient between venous blood and the rest of body for vitamin D_3 and 25(OH)D_3, respectively. Kp_l and Kp_{25l}: distribution coefficient between venous blood and the liver for vitamin D_3 and 25(OH)D_3, respectively. Kp_{fm}: distribution coefficient between venous blood and the fat mass for 25(OH)D_3. Kp_{lm}: distribution coefficient between venous blood and the lean mass for 25(OH)D_3. F_m: fraction of vitamin D_3 metabolised into 25(OH)D_3. CL_H: hepatic clearance rate (L/h). C_{VD}: concentration of hepatic vitamin D_3.

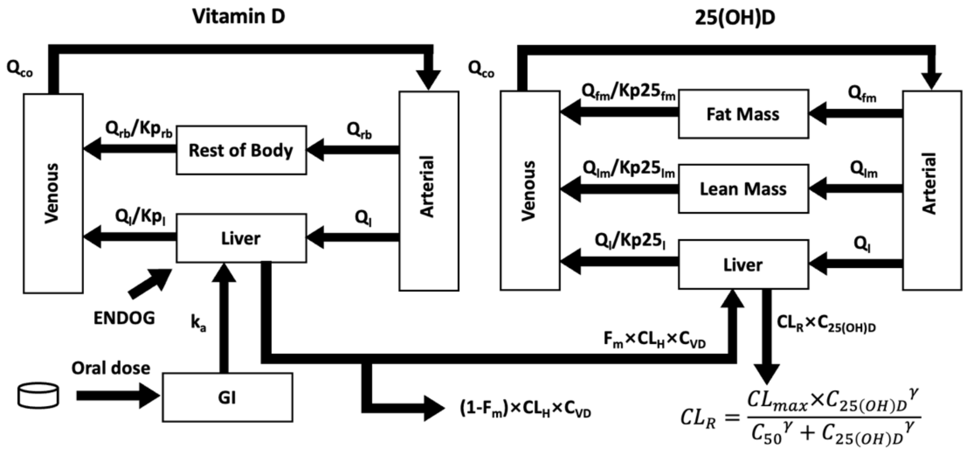

$$ENDOG = \frac{CL_{max} \times D25_{BASE}{}^{\gamma}}{C_{50}{}^{\gamma} + D25_{BASE}{}^{\gamma}} \times D25_{BASE} \times 3$$

$$\frac{dA_{dep}}{dt} = -k_a \times A_{dep}$$

$$\frac{dA_{ven}}{dt} = \frac{Q_l \times A_l}{V_l} \times \frac{1}{K_{pl}} + \frac{Q_{rb} \times A_{rb}}{V_{rb}} \times \frac{1}{K_{prb}} - \frac{Q_{co} \times A_{ven}}{V_{ven}}$$

$$\frac{dA_l}{dt} = -\frac{Q_l \times A_l}{V_l} \times \frac{1}{K_{pl}} + \frac{Q_l \times A_{art}}{V_{art}} - \frac{MCL_H \times A_l}{V_l} \times \frac{1}{K_{pl}} + ENDOG + k_a \times A_{dep}$$

$$\frac{dA25_{ven}}{dt} = \frac{Q_l \times A25_l}{V_l} \times \frac{1}{K_{p25l}} + \frac{Q_{fm} \times A25_{fm}}{V_{fm}} \times \frac{1}{K_{p25fm}} \frac{Q_{lm} \times A25_{lm}}{V_{lm}} \times \frac{1}{K_{p25lm}} - \frac{Q_{co} \times A25_{ven}}{V_{ven}}$$

$$\frac{dA25_l}{dt} = -\frac{Q_l \times A25_l}{V_l} \times \frac{1}{K_{p25l}} + \frac{Q_l \times A25_{art}}{V_{art}} + \frac{MCL_H \times A_l}{V_l} \times \frac{1}{K_{pl}} \times \frac{1}{3} - \frac{CL_{max} \times \left(\frac{A25_l}{V_l} \times \frac{1}{K_{p25l}}\right)^{\gamma}}{{C_{50}}^{\gamma} + \left(\frac{A25_l}{V_l} \times \frac{1}{K_{p25l}}\right)^{\gamma}} \times \frac{A25_l}{V_l} \times \frac{1}{K_{p25l}}$$

$$\frac{dA25_{fm}}{dt} = \frac{Q_{fm} \times A25_{art}}{V_{art}} - \frac{Q_{fm} \times A25_{fm}}{V_{fm}} \times \frac{1}{K_{p25fm}}$$

$$\frac{dA25_{lm}}{dt} = \frac{Q_{lm} \times A25_{art}}{V_{art}} - \frac{Q_{lm} \times A25_{lm}}{V_{lm}} \times \frac{1}{K_{p25lm}}$$

$$\frac{dA25_{art}}{dt} = \frac{Q_{co} \times A25_{ven}}{V_{ven}} - \frac{Q_l \times A25_{art}}{V_{art}} - \frac{(Q_{fm} + Q_{lm}) \times A25_{art}}{V_{art}}$$

Initial conditions:

$$A_{art} = \frac{\frac{Q_{co} \times A_{ven}}{V_{ven}}}{\frac{Q_l + Q_{rb}}{V_{art}}}$$

$$A_{rb} = \frac{A_{art} \times K_{prb} \times V_{rb}}{V_{art}} = \frac{\frac{\frac{Q_{co} \times A_{ven}}{V_{ven}}}{\frac{Q_l + Q_{rb}}{V_{art}}} \times K_{prb} \times V_{rb}}{V_{art}}$$

$$A_{dep}(0) = 0$$

$$A_{ven}(0) = ENDOG \times \frac{V_{ven}}{MCL_H}$$

$$A_l(0) = ENDOG \times \frac{V_l}{MCL_H} \times K_{pl}$$

$$A25_{ven}(0) = D25_{BASE} \times V_{ven}$$

$$A25_l(0) = D25_{BASE} \times V_l \times K_{p25l}$$

$$A25_{fm}(0) = D25_{BASE} \times V_{fm} \times K_{p25fm}$$

$$A25_{lm}(0) = D25_{BASE} \times V_{lm} \times K_{p25lm}$$

$$A25_{art}(0) = D25_{BASE} \times V_{art}$$

Allometric weight scaling on volume of distribution and clearance

$V_T = TV_T \times \frac{WT_{cur}}{30}$

$V_{fm} = FM_0 + (FM_{3Y} - FM_0) \times \frac{time}{24 \times 365 \times 3}$

$$V_{fm} = LM_0+(LM_{3Y}-LM_0)\times\frac{time}{24\times365\times3}$$

$$CL_{MAX} = e^{TCL_{MAX}+\eta_{CL_{MAX}}}\times(\frac{WT_{cur}}{30})^{0.75}$$

$$K_{p25fm}= e^{TKp25_{fm}}$$

Where V_T is the volume of distribution of compartment T, TV_T is typical value for compartment T determined from paediatric bodyweight as presented in **Table S3**, WT is bodyweight, CL_{MAX} is maximum clearance, TCL_{MAX} is typical value of CL_{MAX}, $\eta_{CL_{MAX}}$ is the inter-individual variability of CL_{MAX}.

Figure S4 Serum 25(OH)D concentrations after repeated weekly dosing of 250 μg vitamin D_3.

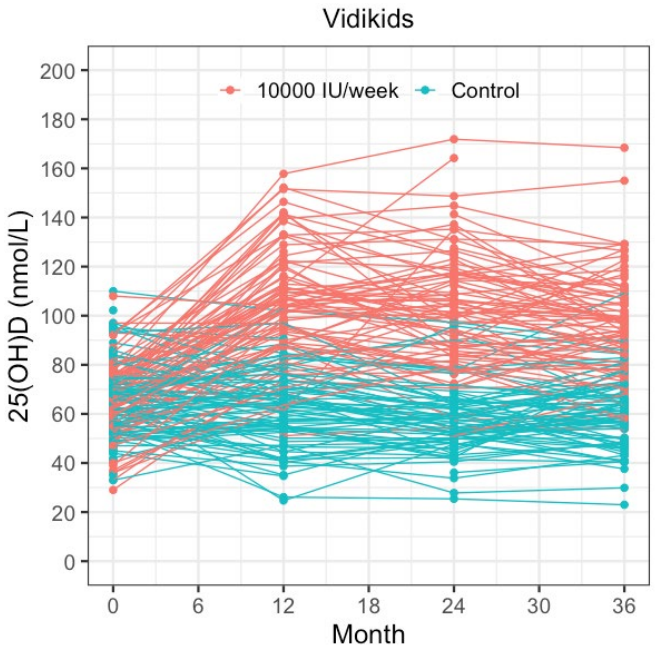

Figure S5. Baseline serum 25(OH)D levels vs. BMI-for-age Z-score for 77 children in the treatment group. The slope of the regression line: -2.644 nmol/L.

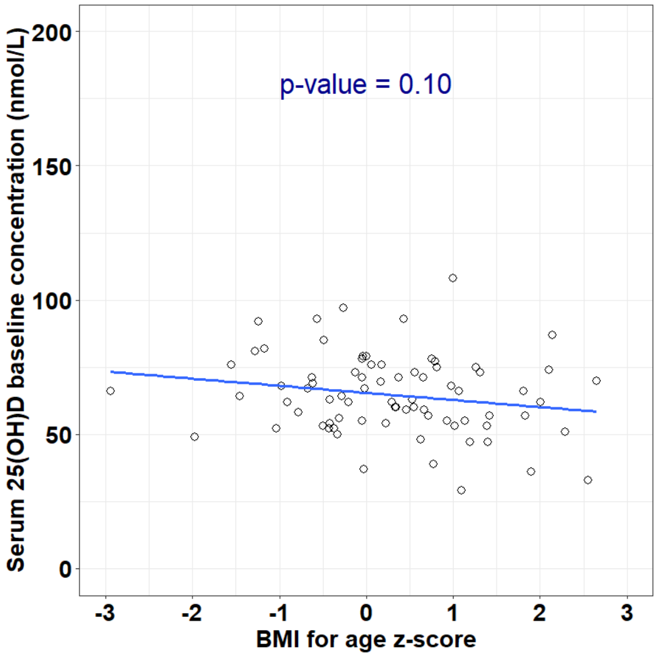

Figure S6. The scatter plot for pharmacokinetic parameters for the final model and various reported covariates.

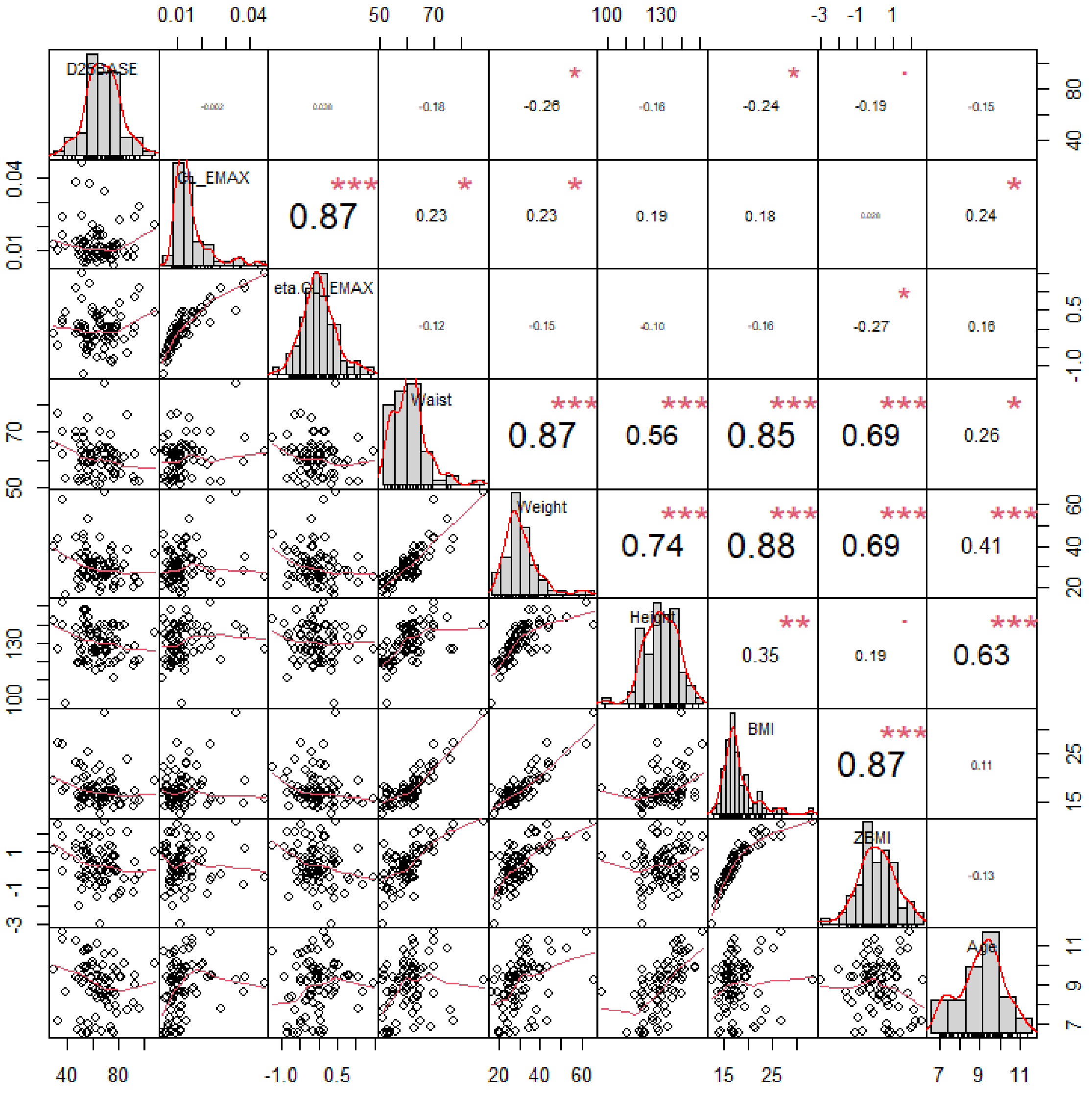

Parameters:

D25BASE, baseline 25OHD; **CL_EMAX,** Maximum clearance (CL_{MAX} in the text); **eta.CL_EMAX,** Between-subject variability of CL_{MAX} ($\eta_{CL_{MAX}}$ in the text); **BMI,** Body Mass Index (kg/m^2); **ZBMI,** BMI-for-age Z-score; **Weight,** Bodyweight in kg; **Waist,** Waist circumference in cm; **Height,** Height in cm; **Age:** Age in years.

Figure S7. Objective function value v Kp25lm. The objective function is fitted at different Kp25lm values: 1, 2, 3, 4, 6, 8.

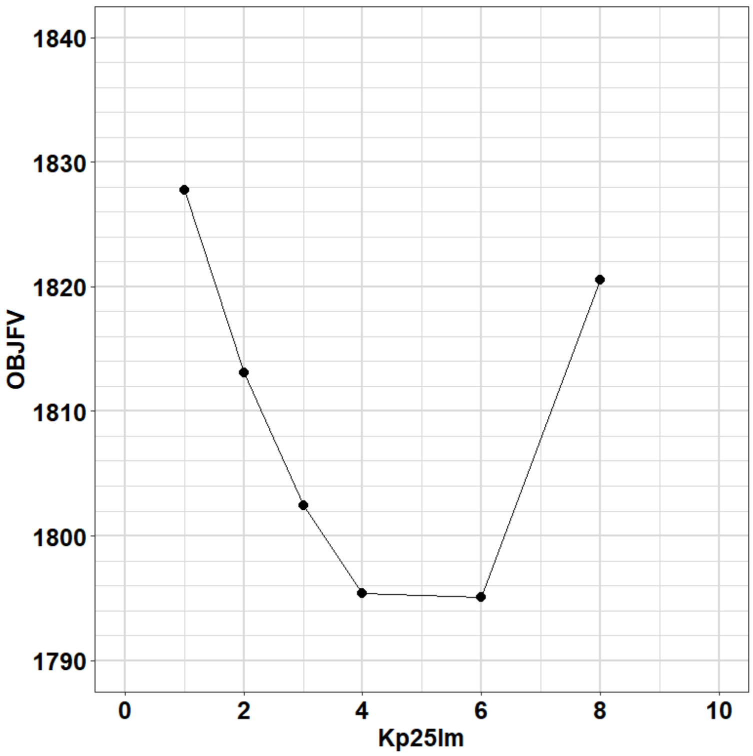

Figure S8. The fitted 77 children. Black solid lines: population predictions. Black dashed lines: individual predictions. Blue dots: observations. Vertical lines: doses.

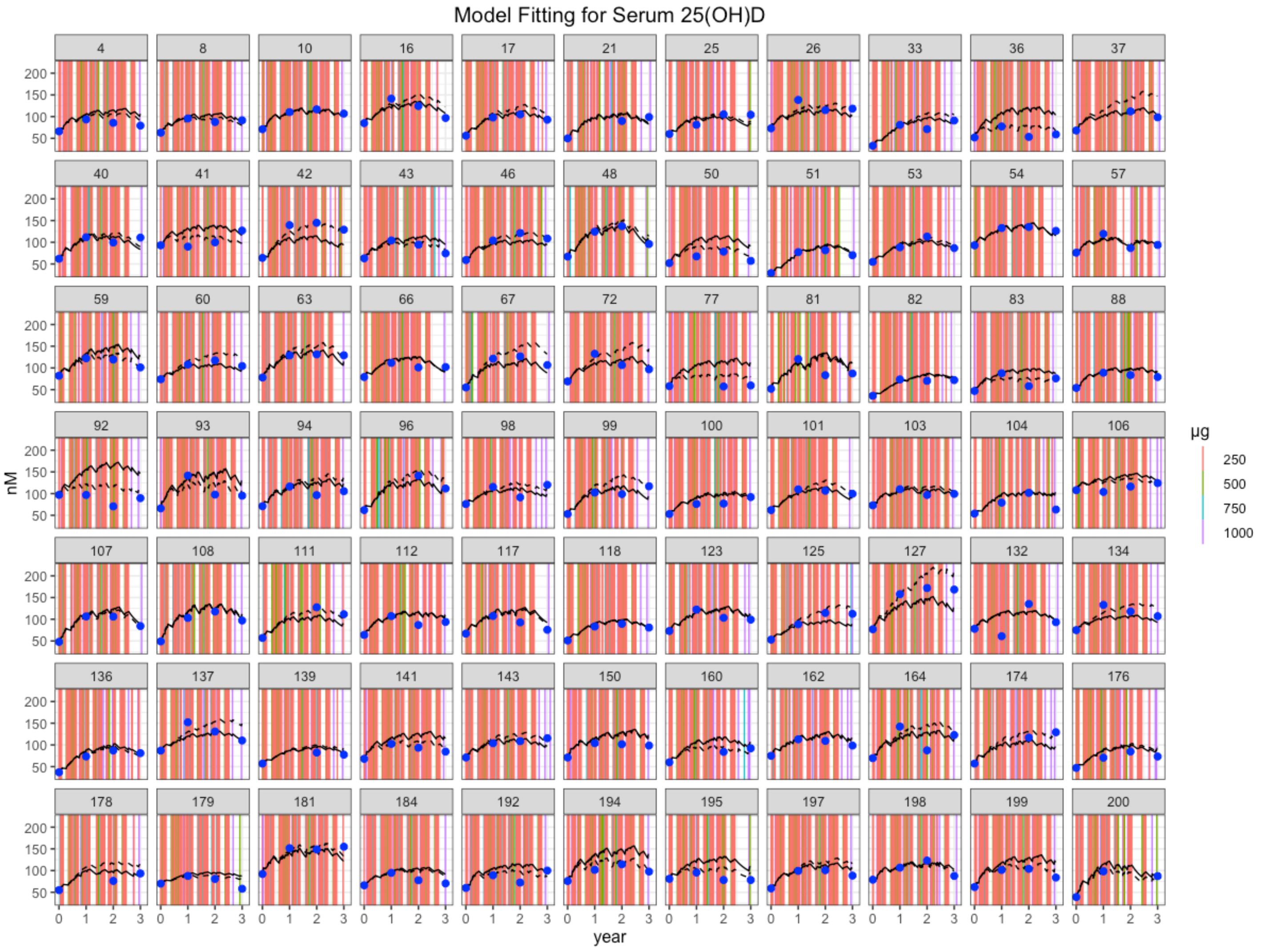

Figure S9. Simulation of serum 25(OH)D concentrations in 454 Cape Town children in the treatment group.

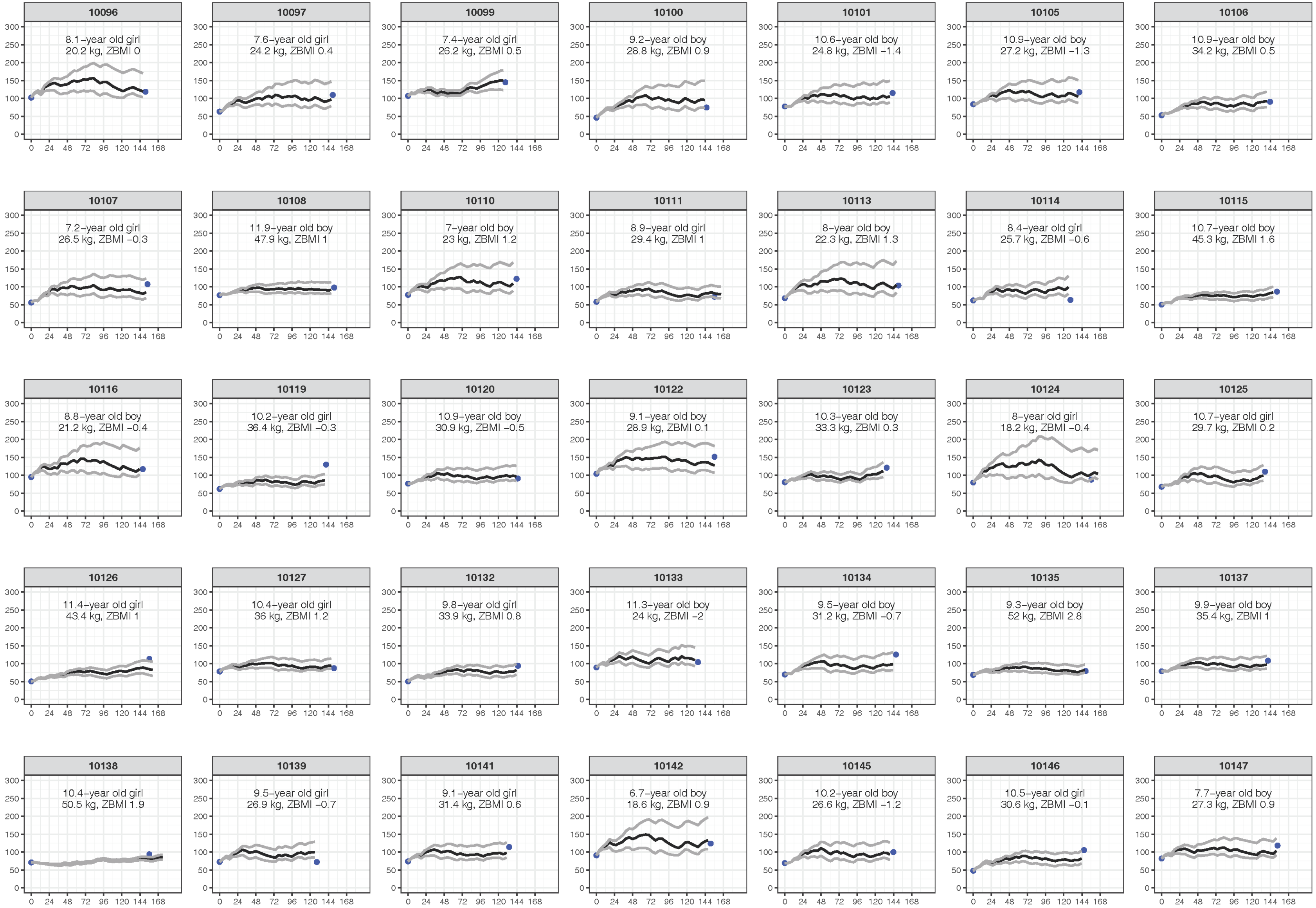

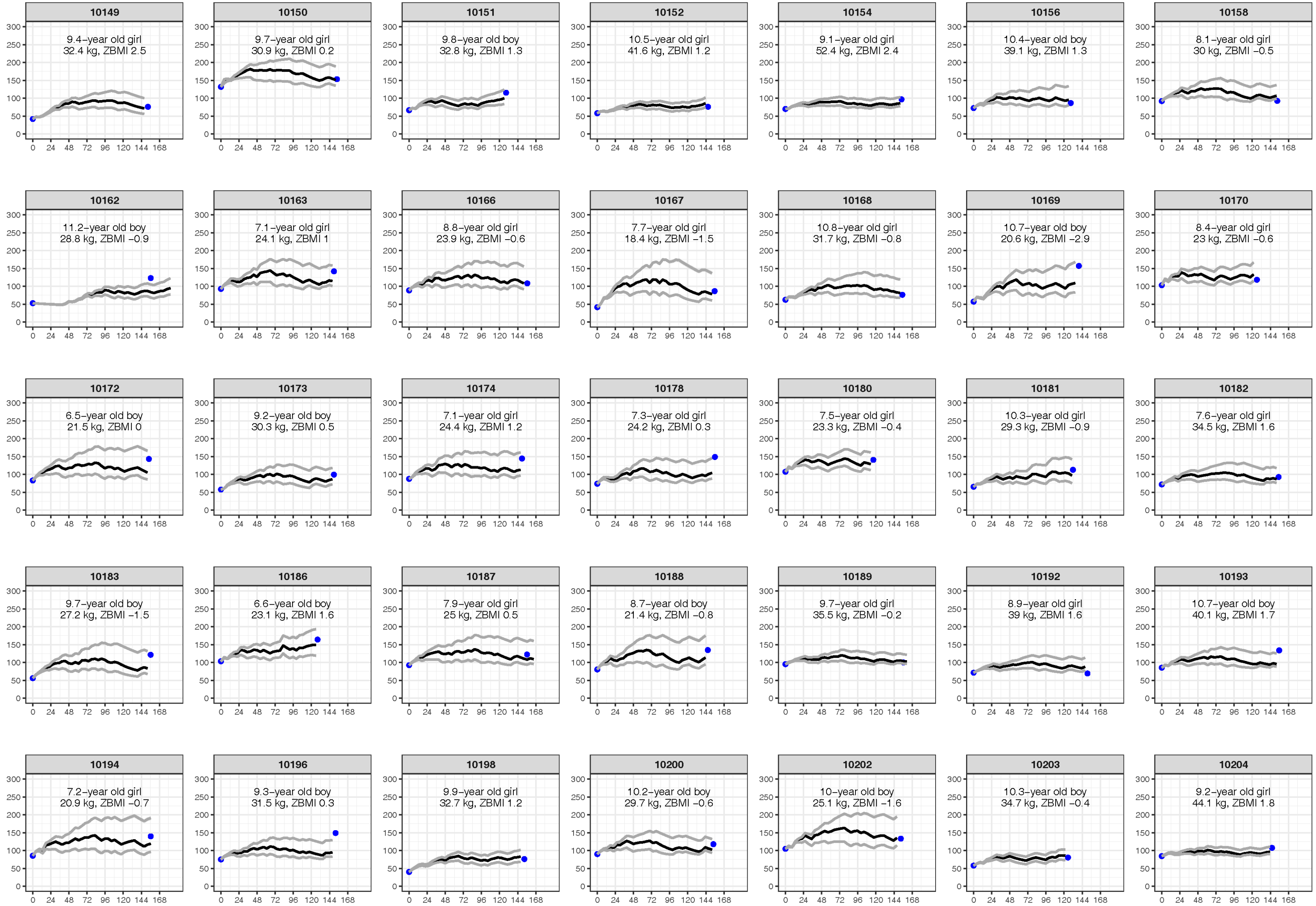
10149
9.4–year old girl
32.4 kg, ZBMI 2.5
10150
9.7–year old girl
30.9 kg, ZBMI 0.2
10151
9.8–year old boy
32.8 kg, ZBMI 1.3
10152
10.5–year old girl
41.6 kg, ZBMI 1.2
10154
9.1–year old girl
52.4 kg, ZBMI 2.4
10156
10.4–year old boy
39.1 kg, ZBMI 1.3
10158
8.1–year old girl
30 kg, ZBMI –0.5
10162
11.2–year old boy
28.8 kg, ZBMI –0.9
10163
7.1–year old girl
24.1 kg, ZBMI 1
10166
8.8–year old girl
23.9 kg, ZBMI –0.6
10167
7.7–year old girl
18.4 kg, ZBMI –1.5
10168
10.8–year old girl
31.7 kg, ZBMI –0.8
10169
10.7–year old boy
20.6 kg, ZBMI –2.9
10170
8.4–year old girl
23 kg, ZBMI –0.6
10172
6.5–year old boy
21.5 kg, ZBMI 0
10173
9.2–year old boy
30.3 kg, ZBMI 0.5
10174
7.1–year old girl
24.4 kg, ZBMI 1.2
10178
7.3–year old girl
24.2 kg, ZBMI 0.3
10180
7.5–year old girl
23.3 kg, ZBMI –0.4
10181
10.3–year old girl
29.3 kg, ZBMI –0.9
10182
7.6–year old girl
34.5 kg, ZBMI 1.6
10183
9.7–year old boy
27.2 kg, ZBMI –1.5
10186
6.6–year old boy
23.1 kg, ZBMI 1.6
10187
7.9–year old girl
25 kg, ZBMI 0.5
10188
8.7–year old boy
21.4 kg, ZBMI –0.8
10189
9.7–year old girl
35.5 kg, ZBMI –0.2
10192
8.9–year old girl
39 kg, ZBMI 1.6
10193
10.7–year old boy
40.1 kg, ZBMI 1.7
10194
7.2–year old girl
20.9 kg, ZBMI –0.7
10196
9.3–year old boy
31.5 kg, ZBMI 0.3
10198
9.9–year old girl
32.7 kg, ZBMI 1.2
10200
10.2–year old boy
29.7 kg, ZBMI –0.6
10202
10–year old boy
25.1 kg, ZBMI –1.6
10203
10.3–year old boy
34.7 kg, ZBMI –0.4
10204
9.2–year old girl
44.1 kg, ZBMI 1.8
0 50 100 150 200 250 300
0 24 48 72 96 120 144 168

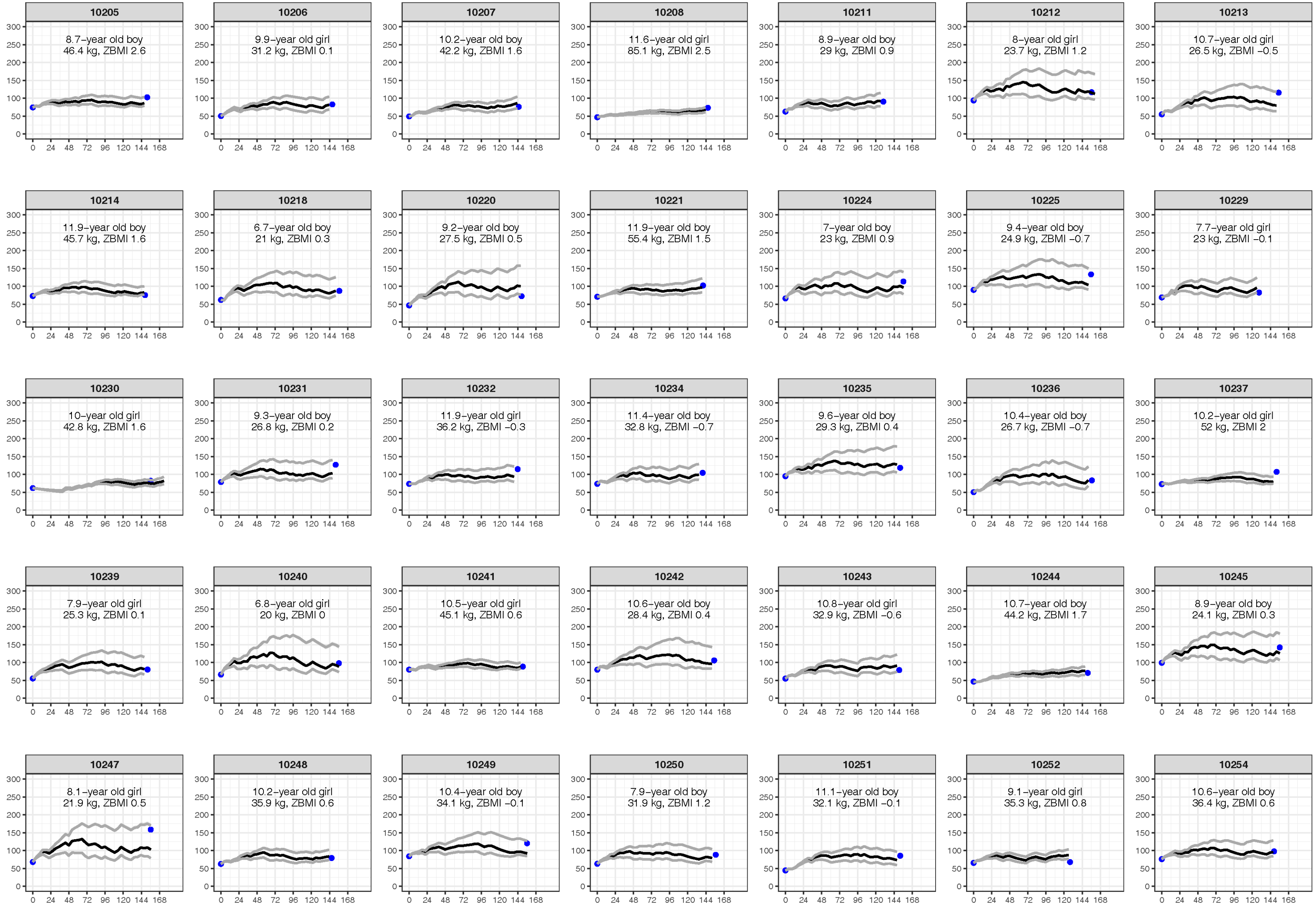
10205
8.7–year old boy
46.4 kg, ZBMI 2.6
10206
9.9–year old girl
31.2 kg, ZBMI 0.1
10207
10.2–year old boy
42.2 kg, ZBMI 1.6
10208
11.6–year old girl
85.1 kg, ZBMI 2.5
10211
8.9–year old boy
29 kg, ZBMI 0.9
10212
8–year old girl
23.7 kg, ZBMI 1.2
10213
10.7–year old girl
26.5 kg, ZBMI –0.5
10214
11.9–year old boy
45.7 kg, ZBMI 1.6
10218
6.7–year old boy
21 kg, ZBMI 0.3
10220
9.2–year old boy
27.5 kg, ZBMI 0.5
10221
11.9–year old boy
55.4 kg, ZBMI 1.5
10224
7–year old boy
23 kg, ZBMI 0.9
10225
9.4–year old boy
24.9 kg, ZBMI –0.7
10229
7.7–year old girl
23 kg, ZBMI –0.1
10230
10–year old girl
42.8 kg, ZBMI 1.6
10231
9.3–year old boy
26.8 kg, ZBMI 0.2
10232
11.9–year old girl
36.2 kg, ZBMI –0.3
10234
11.4–year old boy
32.8 kg, ZBMI –0.7
10235
9.6–year old boy
29.3 kg, ZBMI 0.4
10236
10.4–year old boy
26.7 kg, ZBMI –0.7
10237
10.2–year old girl
52 kg, ZBMI 2
10239
7.9–year old girl
25.3 kg, ZBMI 0.1
10240
6.8–year old girl
20 kg, ZBMI 0
10241
10.5–year old girl
45.1 kg, ZBMI 0.6
10242
10.6–year old boy
28.4 kg, ZBMI 0.4
10243
10.8–year old girl
32.9 kg, ZBMI –0.6
10244
10.7–year old boy
44.2 kg, ZBMI 1.7
10245
8.9–year old boy
24.1 kg, ZBMI 0.3
10247
8.1–year old girl
21.9 kg, ZBMI 0.5
10248
10.2–year old girl
35.9 kg, ZBMI 0.6
10249
10.4–year old boy
34.1 kg, ZBMI –0.1
10250
7.9–year old boy
31.9 kg, ZBMI 1.2
10251
11.1–year old boy
32.1 kg, ZBMI –0.1
10252
9.1–year old girl
35.3 kg, ZBMI 0.8
10254
10.6–year old boy
36.4 kg, ZBMI 0.6
0 50 100 150 200 250 300
0 24 48 72 96 120 144 168

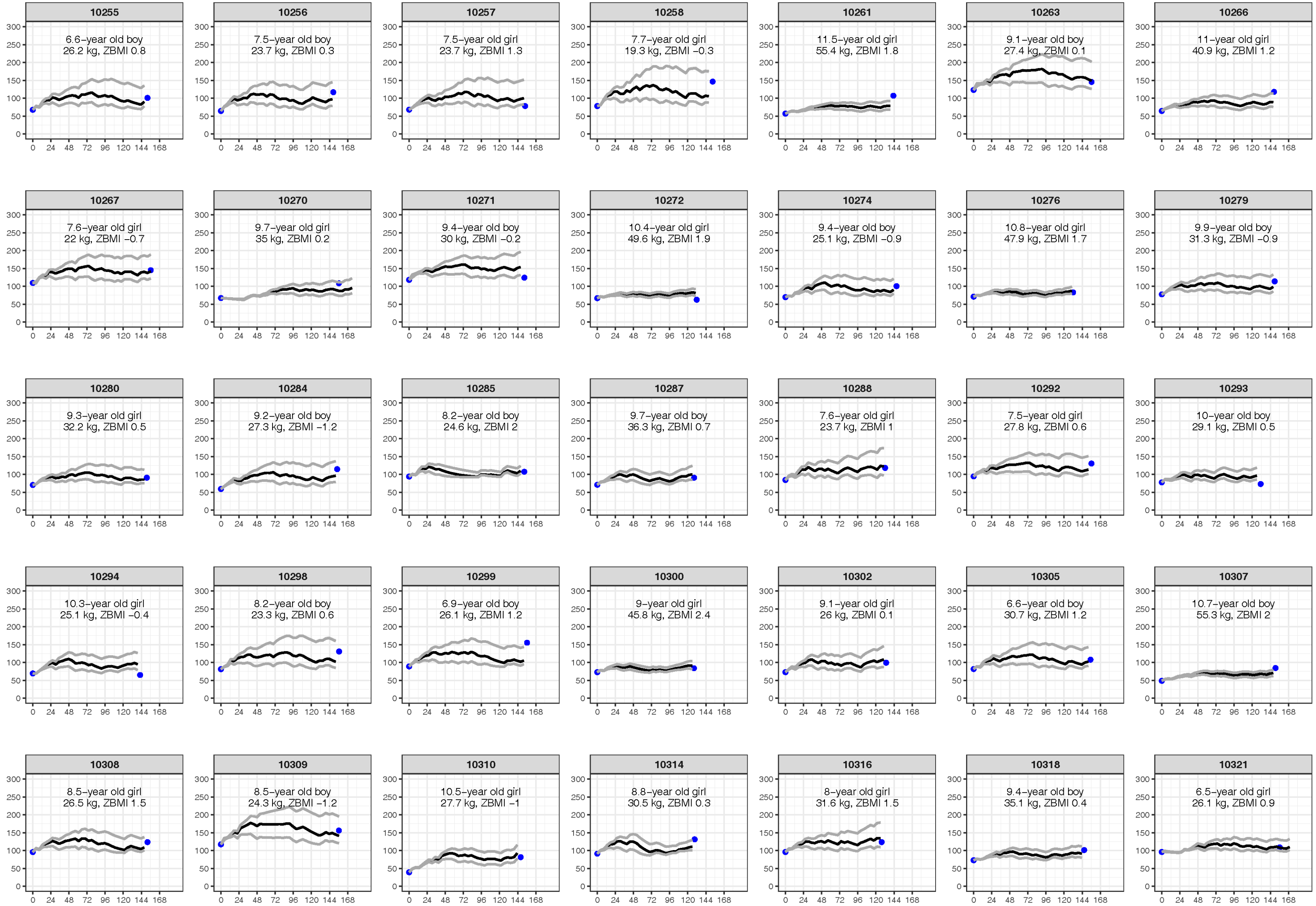
10255
6.6−year old boy
26.2 kg, ZBMI 0.8
10256
7.5−year old boy
23.7 kg, ZBMI 0.3
10257
7.5−year old girl
23.7 kg, ZBMI 1.3
10258
7.7−year old girl
19.3 kg, ZBMI −0.3
10261
11.5−year old girl
55.4 kg, ZBMI 1.8
10263
9.1−year old boy
27.4 kg, ZBMI 0.1
10266
11−year old girl
40.9 kg, ZBMI 1.2
10267
7.6−year old girl
22 kg, ZBMI −0.7
10270
9.7−year old girl
35 kg, ZBMI 0.2
10271
9.4−year old boy
30 kg, ZBMI −0.2
10272
10.4−year old girl
49.6 kg, ZBMI 1.9
10274
9.4−year old boy
25.1 kg, ZBMI −0.9
10276
10.8−year old girl
47.9 kg, ZBMI 1.7
10279
9.9−year old boy
31.3 kg, ZBMI −0.9
10280
9.3−year old girl
32.2 kg, ZBMI 0.5
10284
9.2−year old boy
27.3 kg, ZBMI −1.2
10285
8.2−year old boy
24.6 kg, ZBMI 2
10287
9.7−year old boy
36.3 kg, ZBMI 0.7
10288
7.6−year old girl
23.7 kg, ZBMI 1
10292
7.5−year old girl
27.8 kg, ZBMI 0.6
10293
10−year old boy
29.1 kg, ZBMI 0.5
10294
10.3−year old girl
25.1 kg, ZBMI −0.4
10298
8.2−year old boy
23.3 kg, ZBMI 0.6
10299
6.9−year old boy
26.1 kg, ZBMI 1.2
10300
9−year old girl
45.8 kg, ZBMI 2.4
10302
9.1−year old girl
26 kg, ZBMI 0.1
10305
6.6−year old boy
30.7 kg, ZBMI 1.2
10307
10.7−year old boy
55.3 kg, ZBMI 2
10308
8.5−year old girl
26.5 kg, ZBMI 1.5
10309
8.5−year old boy
24.3 kg, ZBMI −1.2
10310
10.5−year old girl
27.7 kg, ZBMI −1
10314
8.8−year old girl
30.5 kg, ZBMI 0.3
10316
8−year old girl
31.6 kg, ZBMI 1.5
10318
9.4−year old boy
35.1 kg, ZBMI 0.4
10321
6.5−year old girl
26.1 kg, ZBMI 0.9
0 50 100 150 200 250 300
0 24 48 72 96 120 144 168

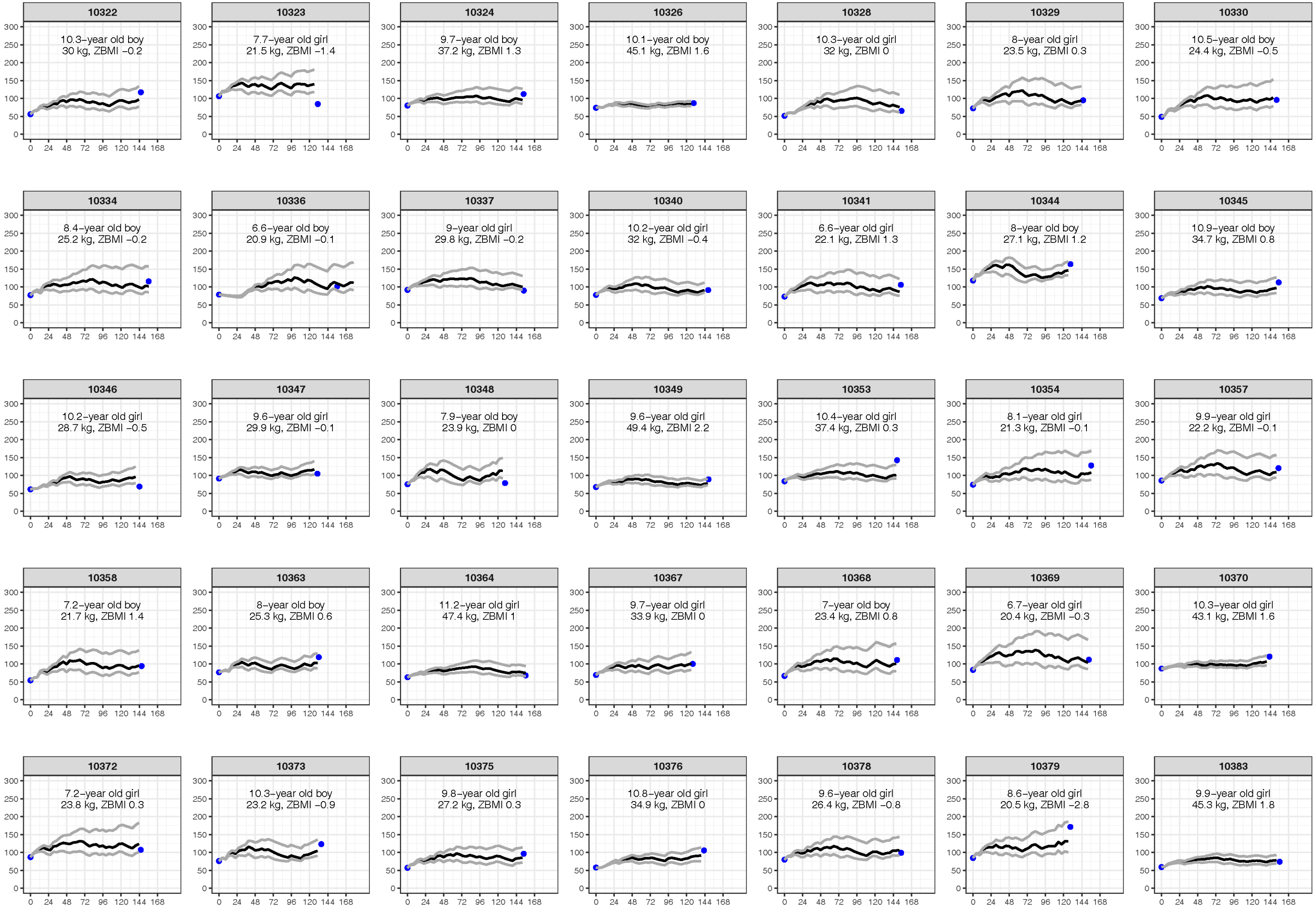
10322
10.3–year old boy
30 kg, ZBMI −0.2
10323
7.7–year old girl
21.5 kg, ZBMI −1.4
10324
9.7–year old boy
37.2 kg, ZBMI 1.3
10326
10.1–year old boy
45.1 kg, ZBMI 1.6
10328
10.3–year old girl
32 kg, ZBMI 0
10329
8–year old girl
23.5 kg, ZBMI 0.3
10330
10.5–year old boy
24.4 kg, ZBMI −0.5
10334
8.4–year old boy
25.2 kg, ZBMI −0.2
10336
6.6–year old boy
20.9 kg, ZBMI −0.1
10337
9–year old girl
29.8 kg, ZBMI −0.2
10340
10.2–year old girl
32 kg, ZBMI −0.4
10341
6.6–year old girl
22.1 kg, ZBMI 1.3
10344
8–year old boy
27.1 kg, ZBMI 1.2
10345
10.9–year old boy
34.7 kg, ZBMI 0.8
10346
10.2–year old girl
28.7 kg, ZBMI −0.5
10347
9.6–year old girl
29.9 kg, ZBMI −0.1
10348
7.9–year old boy
23.9 kg, ZBMI 0
10349
9.6–year old girl
49.4 kg, ZBMI 2.2
10353
10.4–year old girl
37.4 kg, ZBMI 0.3
10354
8.1–year old girl
21.3 kg, ZBMI −0.1
10357
9.9–year old girl
22.2 kg, ZBMI −0.1
10358
7.2–year old boy
21.7 kg, ZBMI 1.4
10363
8–year old boy
25.3 kg, ZBMI 0.6
10364
11.2–year old girl
47.4 kg, ZBMI 1
10367
9.7–year old girl
33.9 kg, ZBMI 0
10368
7–year old boy
23.4 kg, ZBMI 0.8
10369
6.7–year old girl
20.4 kg, ZBMI −0.3
10370
10.3–year old girl
43.1 kg, ZBMI 1.6
10372
7.2–year old girl
23.8 kg, ZBMI 0.3
10373
10.3–year old boy
23.2 kg, ZBMI −0.9
10375
9.8–year old girl
27.2 kg, ZBMI 0.3
10376
10.8–year old girl
34.9 kg, ZBMI 0
10378
9.6–year old girl
26.4 kg, ZBMI −0.8
10379
8.6–year old girl
20.5 kg, ZBMI −2.8
10383
9.9–year old girl
45.3 kg, ZBMI 1.8
0 50 100 150 200 250 300
0 24 48 72 96 120 144 168

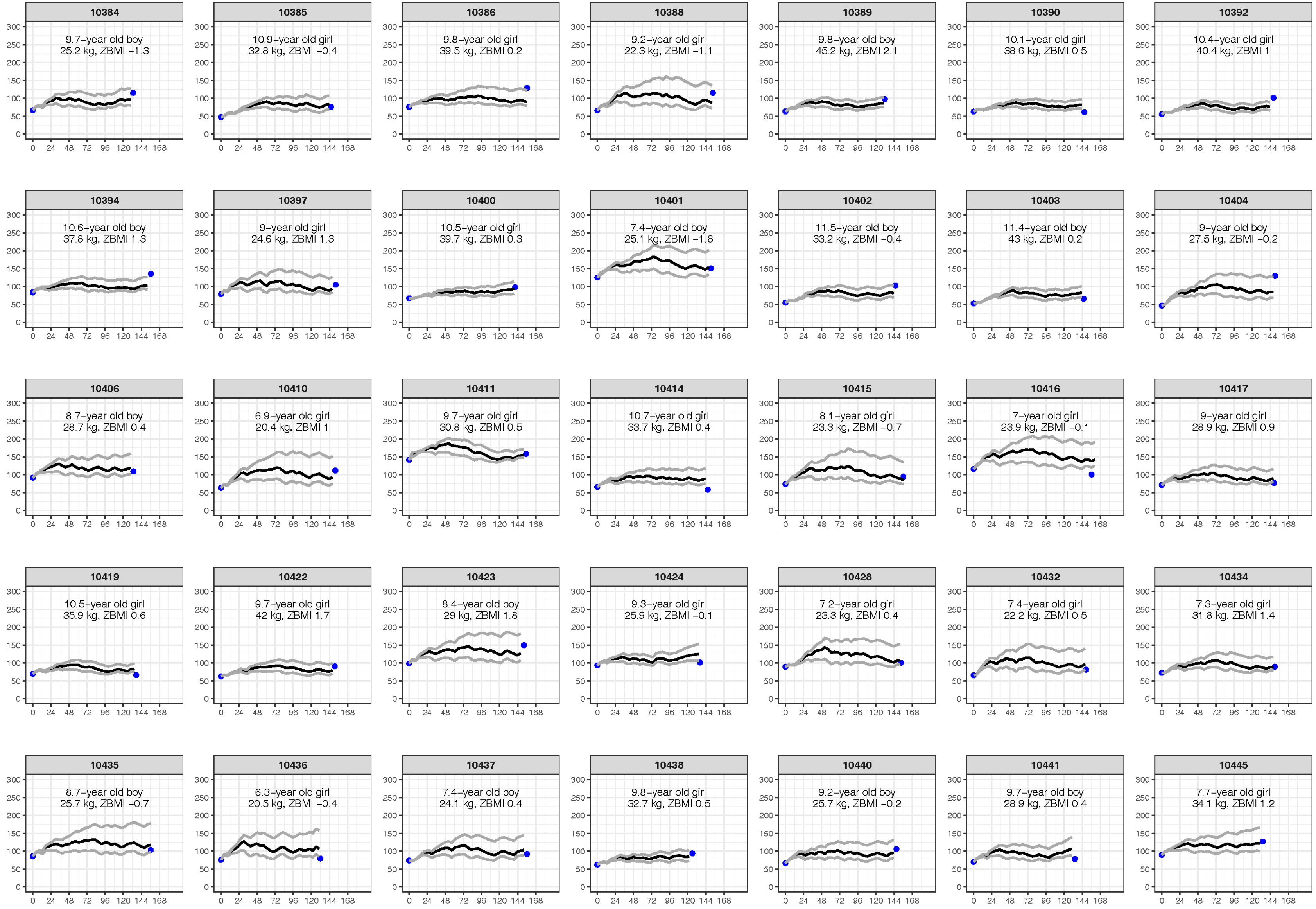
10384
9.7–year old boy
25.2 kg, ZBMI −1.3
10385
10.9–year old girl
32.8 kg, ZBMI −0.4
10386
9.8–year old girl
39.5 kg, ZBMI 0.2
10388
9.2–year old girl
22.3 kg, ZBMI −1.1
10389
9.8–year old boy
45.2 kg, ZBMI 2.1
10390
10.1–year old girl
38.6 kg, ZBMI 0.5
10392
10.4–year old girl
40.4 kg, ZBMI 1
10394
10.6–year old boy
37.8 kg, ZBMI 1.3
10397
9–year old girl
24.6 kg, ZBMI 1.3
10400
10.5–year old girl
39.7 kg, ZBMI 0.3
10401
7.4–year old boy
25.1 kg, ZBMI −1.8
10402
11.5–year old boy
33.2 kg, ZBMI −0.4
10403
11.4–year old boy
43 kg, ZBMI 0.2
10404
9–year old boy
27.5 kg, ZBMI −0.2
10406
8.7–year old boy
28.7 kg, ZBMI 0.4
10410
6.9–year old girl
20.4 kg, ZBMI 1
10411
9.7–year old girl
30.8 kg, ZBMI 0.5
10414
10.7–year old girl
33.7 kg, ZBMI 0.4
10415
8.1–year old girl
23.3 kg, ZBMI −0.7
10416
7–year old girl
23.9 kg, ZBMI −0.1
10417
9–year old girl
28.9 kg, ZBMI 0.9
10419
10.5–year old girl
35.9 kg, ZBMI 0.6
10422
9.7–year old girl
42 kg, ZBMI 1.7
10423
8.4–year old boy
29 kg, ZBMI 1.8
10424
9.3–year old girl
25.9 kg, ZBMI −0.1
10428
7.2–year old girl
23.3 kg, ZBMI 0.4
10432
7.4–year old girl
22.2 kg, ZBMI 0.5
10434
7.3–year old girl
31.8 kg, ZBMI 1.4
10435
8.7–year old boy
25.7 kg, ZBMI −0.7
10436
6.3–year old girl
20.5 kg, ZBMI −0.4
10437
7.4–year old boy
24.1 kg, ZBMI 0.4
10438
9.8–year old girl
32.7 kg, ZBMI 0.5
10440
9.2–year old boy
25.7 kg, ZBMI −0.2
10441
9.7–year old boy
28.9 kg, ZBMI 0.4
10445
7.7–year old girl
34.1 kg, ZBMI 1.2
0 50 100 150 200 250 300
0 24 48 72 96 120 144 168

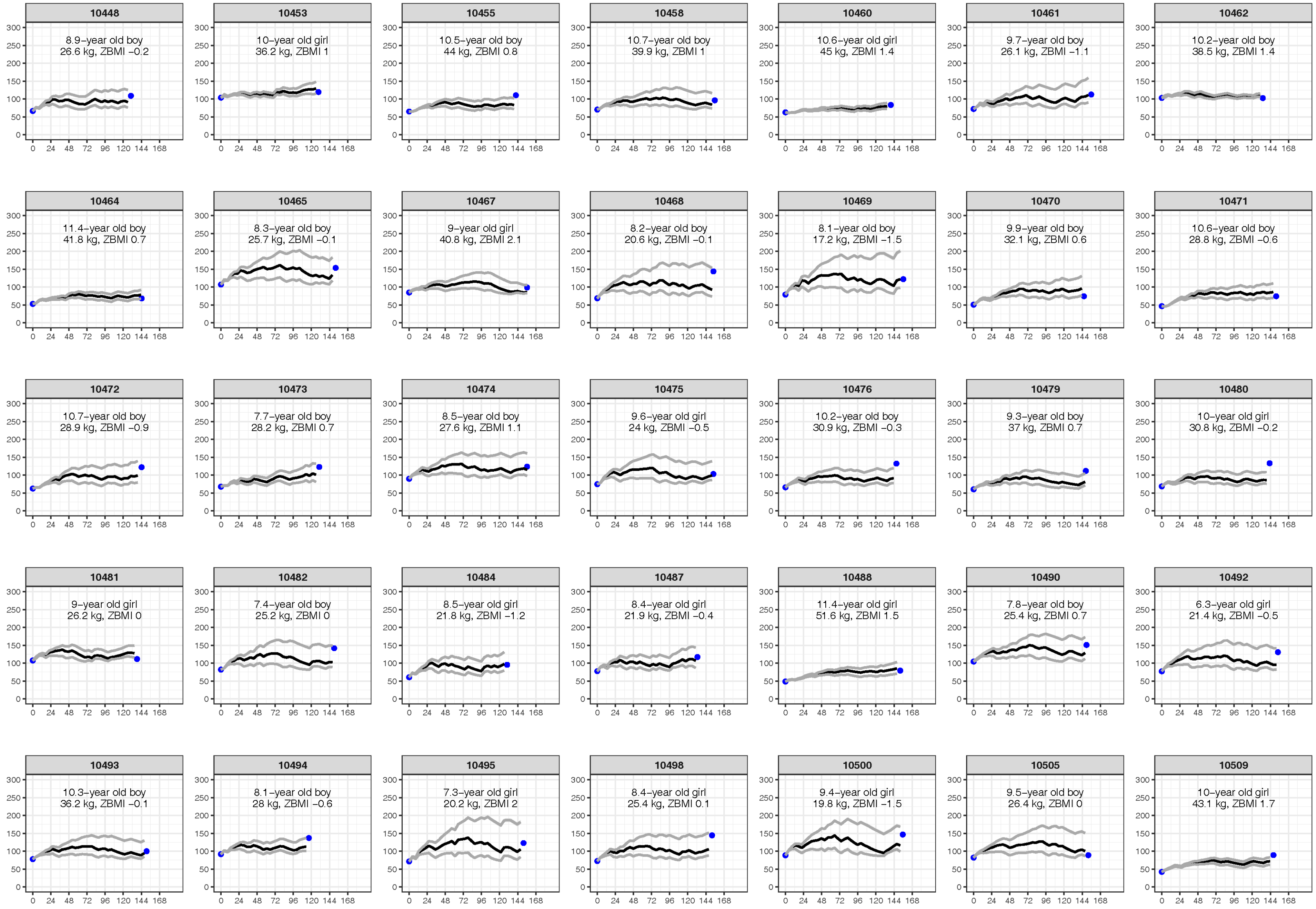
10448
8.9–year old boy
26.6 kg, ZBMI −0.2
10453
10–year old girl
36.2 kg, ZBMI 1
10455
10.5–year old boy
44 kg, ZBMI 0.8
10458
10.7–year old boy
39.9 kg, ZBMI 1
10460
10.6–year old girl
45 kg, ZBMI 1.4
10461
9.7–year old boy
26.1 kg, ZBMI −1.1
10462
10.2–year old boy
38.5 kg, ZBMI 1.4
10464
11.4–year old boy
41.8 kg, ZBMI 0.7
10465
8.3–year old boy
25.7 kg, ZBMI −0.1
10467
9–year old girl
40.8 kg, ZBMI 2.1
10468
8.2–year old boy
20.6 kg, ZBMI −0.1
10469
8.1–year old boy
17.2 kg, ZBMI −1.5
10470
9.9–year old boy
32.1 kg, ZBMI 0.6
10471
10.6–year old boy
28.8 kg, ZBMI −0.6
10472
10.7–year old boy
28.9 kg, ZBMI −0.9
10473
7.7–year old boy
28.2 kg, ZBMI 0.7
10474
8.5–year old boy
27.6 kg, ZBMI 1.1
10475
9.6–year old girl
24 kg, ZBMI −0.5
10476
10.2–year old boy
30.9 kg, ZBMI −0.3
10479
9.3–year old boy
37 kg, ZBMI 0.7
10480
10–year old girl
30.8 kg, ZBMI −0.2
10481
9–year old girl
26.2 kg, ZBMI 0
10482
7.4–year old boy
25.2 kg, ZBMI 0
10484
8.5–year old girl
21.8 kg, ZBMI −1.2
10487
8.4–year old girl
21.9 kg, ZBMI −0.4
10488
11.4–year old girl
51.6 kg, ZBMI 1.5
10490
7.8–year old boy
25.4 kg, ZBMI 0.7
10492
6.3–year old girl
21.4 kg, ZBMI −0.5
10493
10.3–year old boy
36.2 kg, ZBMI −0.1
10494
8.1–year old boy
28 kg, ZBMI −0.6
10495
7.3–year old girl
20.2 kg, ZBMI 2
10498
8.4–year old girl
25.4 kg, ZBMI 0.1
10500
9.4–year old girl
19.8 kg, ZBMI −1.5
10505
9.5–year old boy
26.4 kg, ZBMI 0
10509
10–year old girl
43.1 kg, ZBMI 1.7
0 24 48 72 96 120 144 168
0 50 100 150 200 250 300

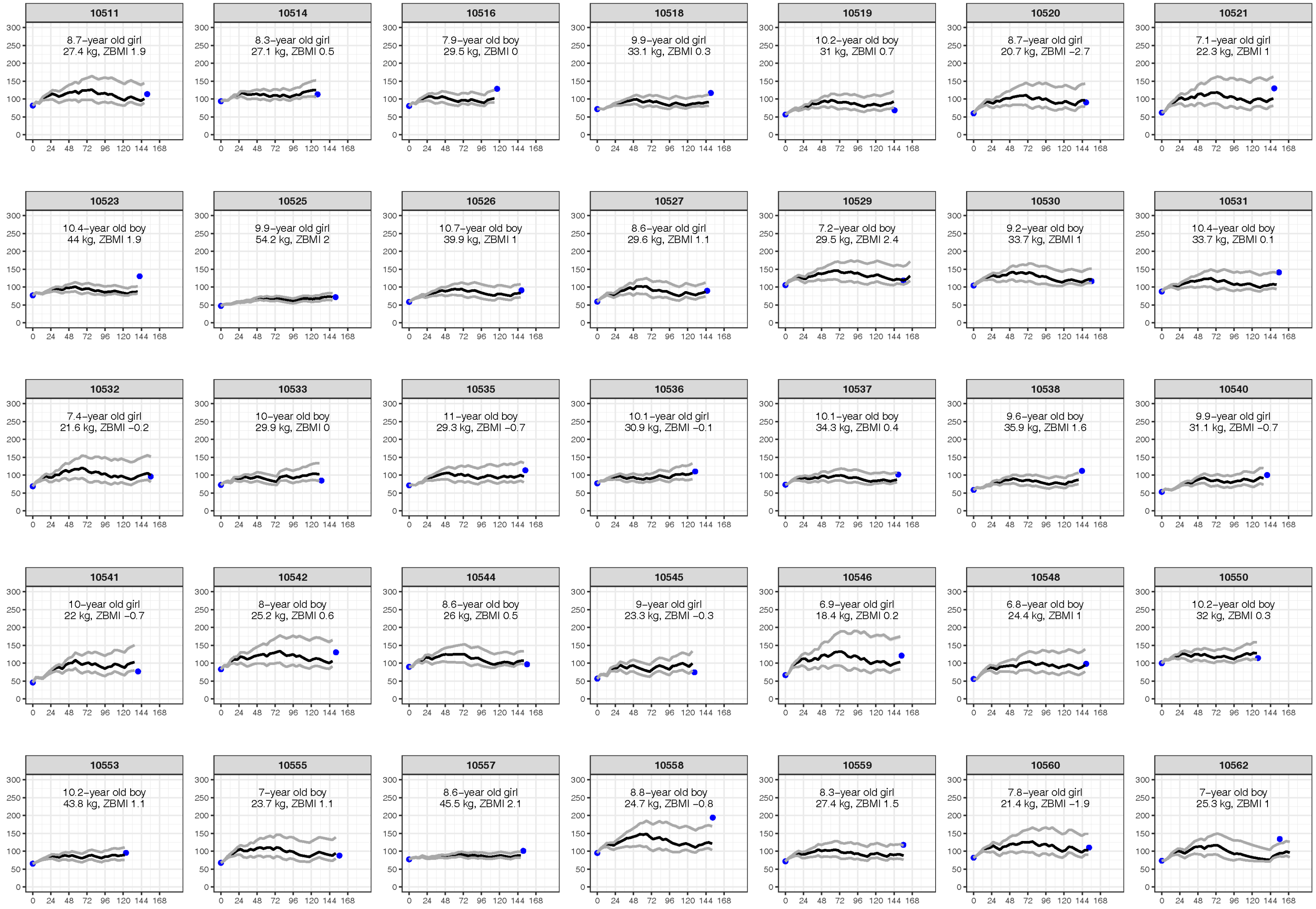

10511
8.7−year old girl
27.4 kg, ZBMI 1.9
10514
8.3−year old girl
27.1 kg, ZBMI 0.5
10516
7.9−year old boy
29.5 kg, ZBMI 0
10518
9.9−year old girl
33.1 kg, ZBMI 0.3
10519
10.2−year old boy
31 kg, ZBMI 0.7
10520
8.7−year old girl
20.7 kg, ZBMI −2.7
10521
7.1−year old girl
22.3 kg, ZBMI 1
10523
10.4−year old boy
44 kg, ZBMI 1.9
10525
9.9−year old girl
54.2 kg, ZBMI 2
10526
10.7−year old boy
39.9 kg, ZBMI 1
10527
8.6−year old girl
29.6 kg, ZBMI 1.1
10529
7.2−year old boy
29.5 kg, ZBMI 2.4
10530
9.2−year old boy
33.7 kg, ZBMI 1
10531
10.4−year old boy
33.7 kg, ZBMI 0.1
10532
7.4−year old girl
21.6 kg, ZBMI −0.2
10533
10−year old boy
29.9 kg, ZBMI 0
10535
11−year old boy
29.3 kg, ZBMI −0.7
10536
10.1−year old girl
30.9 kg, ZBMI −0.1
10537
10.1−year old boy
34.3 kg, ZBMI 0.4
10538
9.6−year old boy
35.9 kg, ZBMI 1.6
10540
9.9−year old girl
31.1 kg, ZBMI −0.7
10541
10−year old girl
22 kg, ZBMI −0.7
10542
8−year old boy
25.2 kg, ZBMI 0.6
10544
8.6−year old boy
26 kg, ZBMI 0.5
10545
9−year old girl
23.3 kg, ZBMI −0.3
10546
6.9−year old girl
18.4 kg, ZBMI 0.2
10548
6.8−year old boy
24.4 kg, ZBMI 1
10550
10.2−year old boy
32 kg, ZBMI 0.3
10553
10.2−year old boy
43.8 kg, ZBMI 1.1
10555
7−year old boy
23.7 kg, ZBMI 1.1
10557
8.6−year old girl
45.5 kg, ZBMI 2.1
10558
8.8−year old boy
24.7 kg, ZBMI −0.8
10559
8.3−year old girl
27.4 kg, ZBMI 1.5
10560
7.8−year old girl
21.4 kg, ZBMI −1.9
10562
7−year old boy
25.3 kg, ZBMI 1
0 50 100 150 200 250 300
0 24 48 72 96 120 144 168

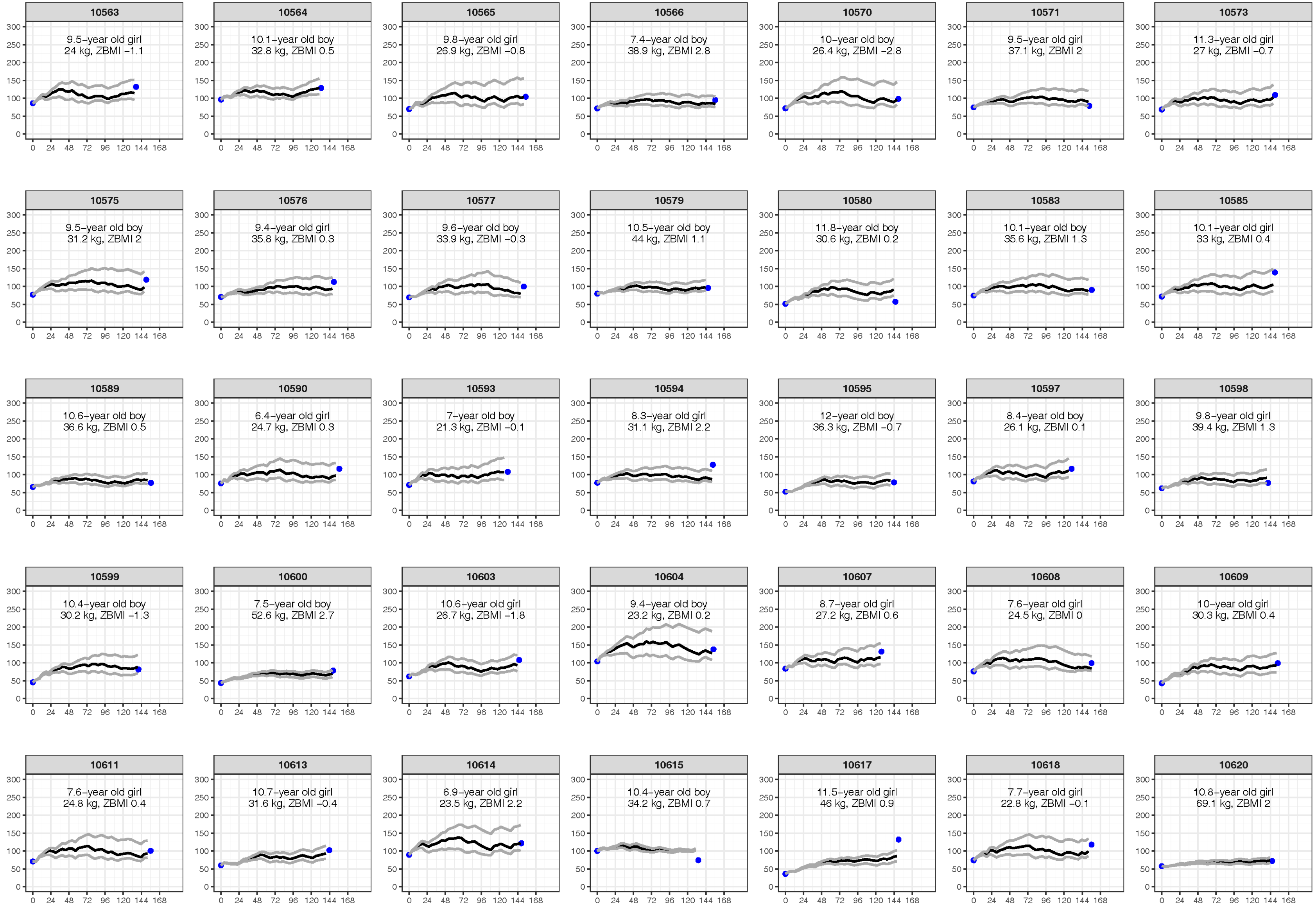
10563
9.5–year old girl
24 kg, ZBMI −1.1
10564
10.1–year old boy
32.8 kg, ZBMI 0.5
10565
9.8–year old girl
26.9 kg, ZBMI −0.8
10566
7.4–year old boy
38.9 kg, ZBMI 2.8
10570
10–year old boy
26.4 kg, ZBMI −2.8
10571
9.5–year old girl
37.1 kg, ZBMI 2
10573
11.3–year old girl
27 kg, ZBMI −0.7
10575
9.5–year old boy
31.2 kg, ZBMI 2
10576
9.4–year old girl
35.8 kg, ZBMI 0.3
10577
9.6–year old boy
33.9 kg, ZBMI −0.3
10579
10.5–year old boy
44 kg, ZBMI 1.1
10580
11.8–year old boy
30.6 kg, ZBMI 0.2
10583
10.1–year old boy
35.6 kg, ZBMI 1.3
10585
10.1–year old girl
33 kg, ZBMI 0.4
10589
10.6–year old boy
36.6 kg, ZBMI 0.5
10590
6.4–year old girl
24.7 kg, ZBMI 0.3
10593
7–year old boy
21.3 kg, ZBMI −0.1
10594
8.3–year old girl
31.1 kg, ZBMI 2.2
10595
12–year old boy
36.3 kg, ZBMI −0.7
10597
8.4–year old boy
26.1 kg, ZBMI 0.1
10598
9.8–year old girl
39.4 kg, ZBMI 1.3
10599
10.4–year old boy
30.2 kg, ZBMI −1.3
10600
7.5–year old boy
52.6 kg, ZBMI 2.7
10603
10.6–year old girl
26.7 kg, ZBMI −1.8
10604
9.4–year old boy
23.2 kg, ZBMI 0.2
10607
8.7–year old girl
27.2 kg, ZBMI 0.6
10608
7.6–year old girl
24.5 kg, ZBMI 0
10609
10–year old girl
30.3 kg, ZBMI 0.4
10611
7.6–year old girl
24.8 kg, ZBMI 0.4
10613
10.7–year old girl
31.6 kg, ZBMI −0.4
10614
6.9–year old girl
23.5 kg, ZBMI 2.2
10615
10.4–year old boy
34.2 kg, ZBMI 0.7
10617
11.5–year old girl
46 kg, ZBMI 0.9
10618
7.7–year old girl
22.8 kg, ZBMI −0.1
10620
10.8–year old girl
69.1 kg, ZBMI 2
0 50 100 150 200 250 300
0 24 48 72 96 120 144 168

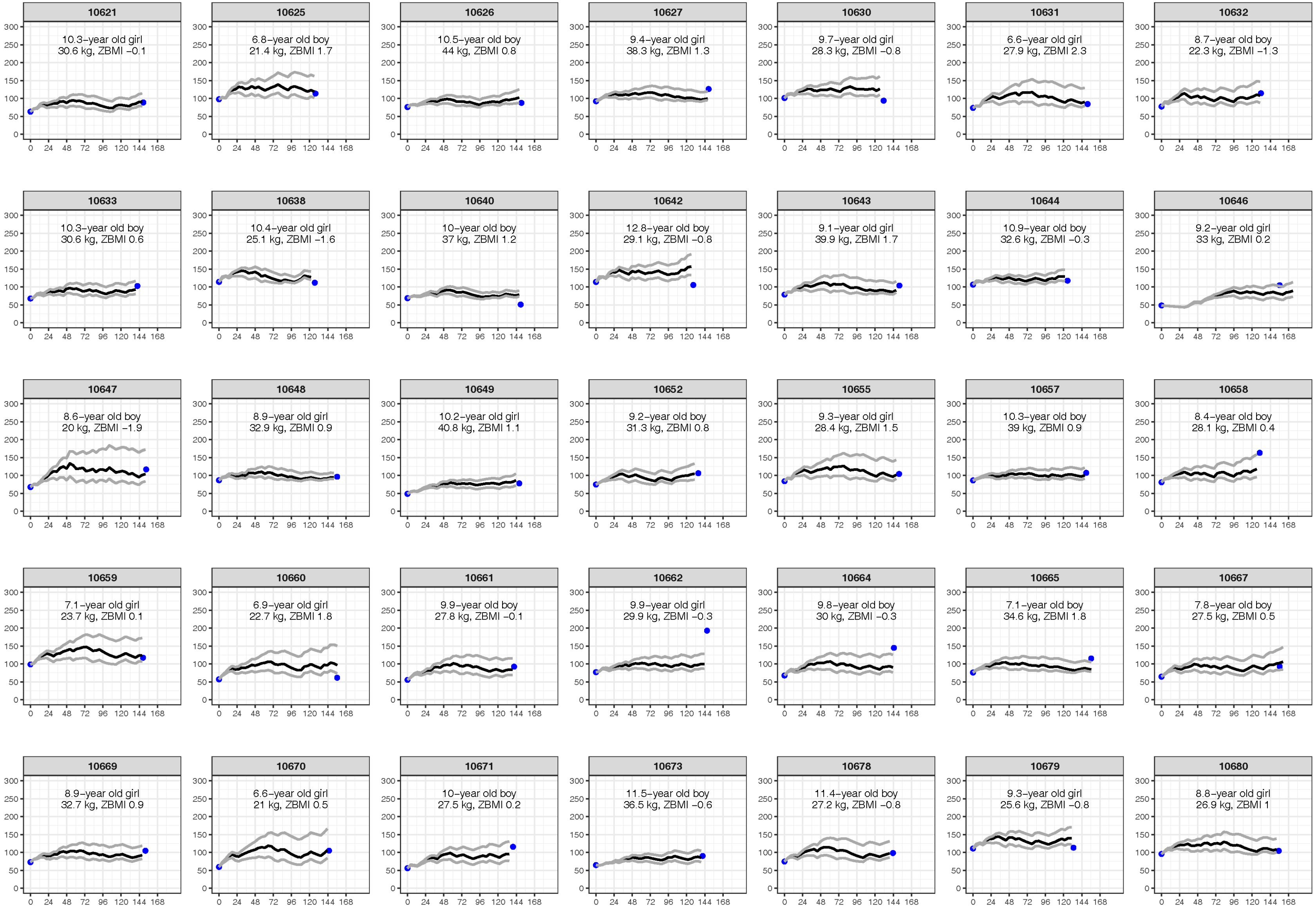
10621
10.3−year old girl
30.6 kg, ZBMI −0.1
10625
6.8−year old boy
21.4 kg, ZBMI 1.7
10626
10.5−year old boy
44 kg, ZBMI 0.8
10627
9.4−year old girl
38.3 kg, ZBMI 1.3
10630
9.7−year old girl
28.3 kg, ZBMI −0.8
10631
6.6−year old girl
27.9 kg, ZBMI 2.3
10632
8.7−year old boy
22.3 kg, ZBMI −1.3
10633
10.3−year old boy
30.6 kg, ZBMI 0.6
10638
10.4−year old girl
25.1 kg, ZBMI −1.6
10640
10−year old boy
37 kg, ZBMI 1.2
10642
12.8−year old boy
29.1 kg, ZBMI −0.8
10643
9.1−year old girl
39.9 kg, ZBMI 1.7
10644
10.9−year old boy
32.6 kg, ZBMI −0.3
10646
9.2−year old girl
33 kg, ZBMI 0.2
10647
8.6−year old boy
20 kg, ZBMI −1.9
10648
8.9−year old girl
32.9 kg, ZBMI 0.9
10649
10.2−year old girl
40.8 kg, ZBMI 1.1
10652
9.2−year old boy
31.3 kg, ZBMI 0.8
10655
9.3−year old girl
28.4 kg, ZBMI 1.5
10657
10.3−year old boy
39 kg, ZBMI 0.9
10658
8.4−year old boy
28.1 kg, ZBMI 0.4
10659
7.1−year old girl
23.7 kg, ZBMI 0.1
10660
6.9−year old girl
22.7 kg, ZBMI 1.8
10661
9.9−year old boy
27.8 kg, ZBMI −0.1
10662
9.9−year old girl
29.9 kg, ZBMI −0.3
10664
9.8−year old boy
30 kg, ZBMI −0.3
10665
7.1−year old boy
34.6 kg, ZBMI 1.8
10667
7.8−year old boy
27.5 kg, ZBMI 0.5
10669
8.9−year old girl
32.7 kg, ZBMI 0.9
10670
6.6−year old girl
21 kg, ZBMI 0.5
10671
10−year old boy
27.5 kg, ZBMI 0.2
10673
11.5−year old boy
36.5 kg, ZBMI −0.6
10678
11.4−year old boy
27.2 kg, ZBMI −0.8
10679
9.3−year old girl
25.6 kg, ZBMI −0.8
10680
8.8−year old girl
26.9 kg, ZBMI 1
0 50 100 150 200 250 300
0 24 48 72 96 120 144 168

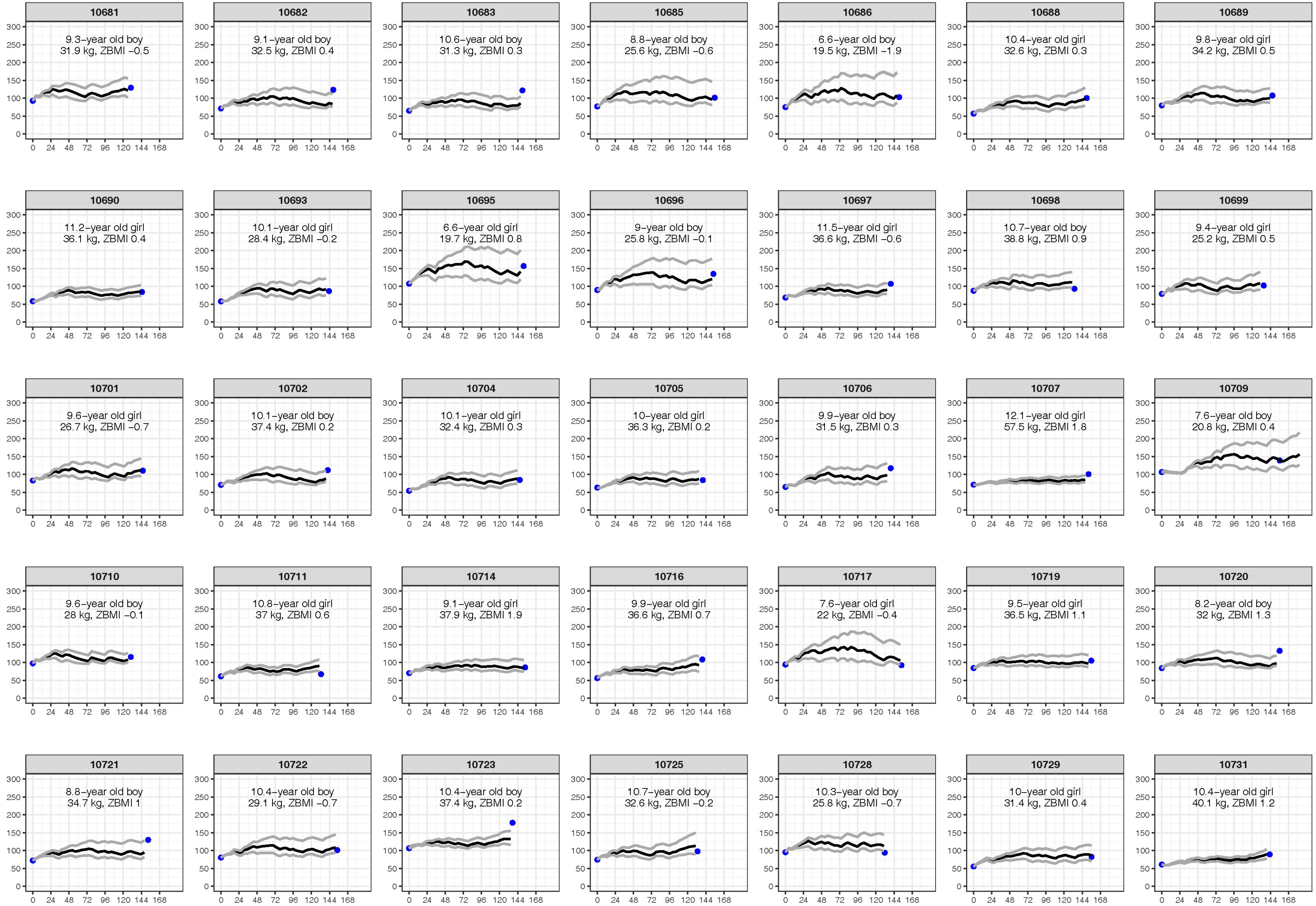

10681
9.3–year old boy
31.9 kg, ZBMI −0.5
10682
9.1–year old boy
32.5 kg, ZBMI 0.4
10683
10.6–year old boy
31.3 kg, ZBMI 0.3
10685
8.8–year old boy
25.6 kg, ZBMI −0.6
10686
6.6–year old boy
19.5 kg, ZBMI −1.9
10688
10.4–year old girl
32.6 kg, ZBMI 0.3
10689
9.8–year old girl
34.2 kg, ZBMI 0.5
10690
11.2–year old girl
36.1 kg, ZBMI 0.4
10693
10.1–year old girl
28.4 kg, ZBMI −0.2
10695
6.6–year old girl
19.7 kg, ZBMI 0.8
10696
9–year old boy
25.8 kg, ZBMI −0.1
10697
11.5–year old girl
36.6 kg, ZBMI −0.6
10698
10.7–year old boy
38.8 kg, ZBMI 0.9
10699
9.4–year old girl
25.2 kg, ZBMI 0.5
10701
9.6–year old girl
26.7 kg, ZBMI −0.7
10702
10.1–year old boy
37.4 kg, ZBMI 0.2
10704
10.1–year old girl
32.4 kg, ZBMI 0.3
10705
10–year old girl
36.3 kg, ZBMI 0.2
10706
9.9–year old boy
31.5 kg, ZBMI 0.3
10707
12.1–year old girl
57.5 kg, ZBMI 1.8
10709
7.6–year old boy
20.8 kg, ZBMI 0.4
10710
9.6–year old boy
28 kg, ZBMI −0.1
10711
10.8–year old girl
37 kg, ZBMI 0.6
10714
9.1–year old girl
37.9 kg, ZBMI 1.9
10716
9.9–year old girl
36.6 kg, ZBMI 0.7
10717
7.6–year old girl
22 kg, ZBMI −0.4
10719
9.5–year old girl
36.5 kg, ZBMI 1.1
10720
8.2–year old boy
32 kg, ZBMI 1.3
10721
8.8–year old boy
34.7 kg, ZBMI 1
10722
10.4–year old boy
29.1 kg, ZBMI −0.7
10723
10.4–year old boy
37.4 kg, ZBMI 0.2
10725
10.7–year old boy
32.6 kg, ZBMI −0.2
10728
10.3–year old boy
25.8 kg, ZBMI −0.7
10729
10–year old girl
31.4 kg, ZBMI 0.4
10731
10.4–year old girl
40.1 kg, ZBMI 1.2
0 50 100 150 200 250 300
0 24 48 72 96 120 144 168

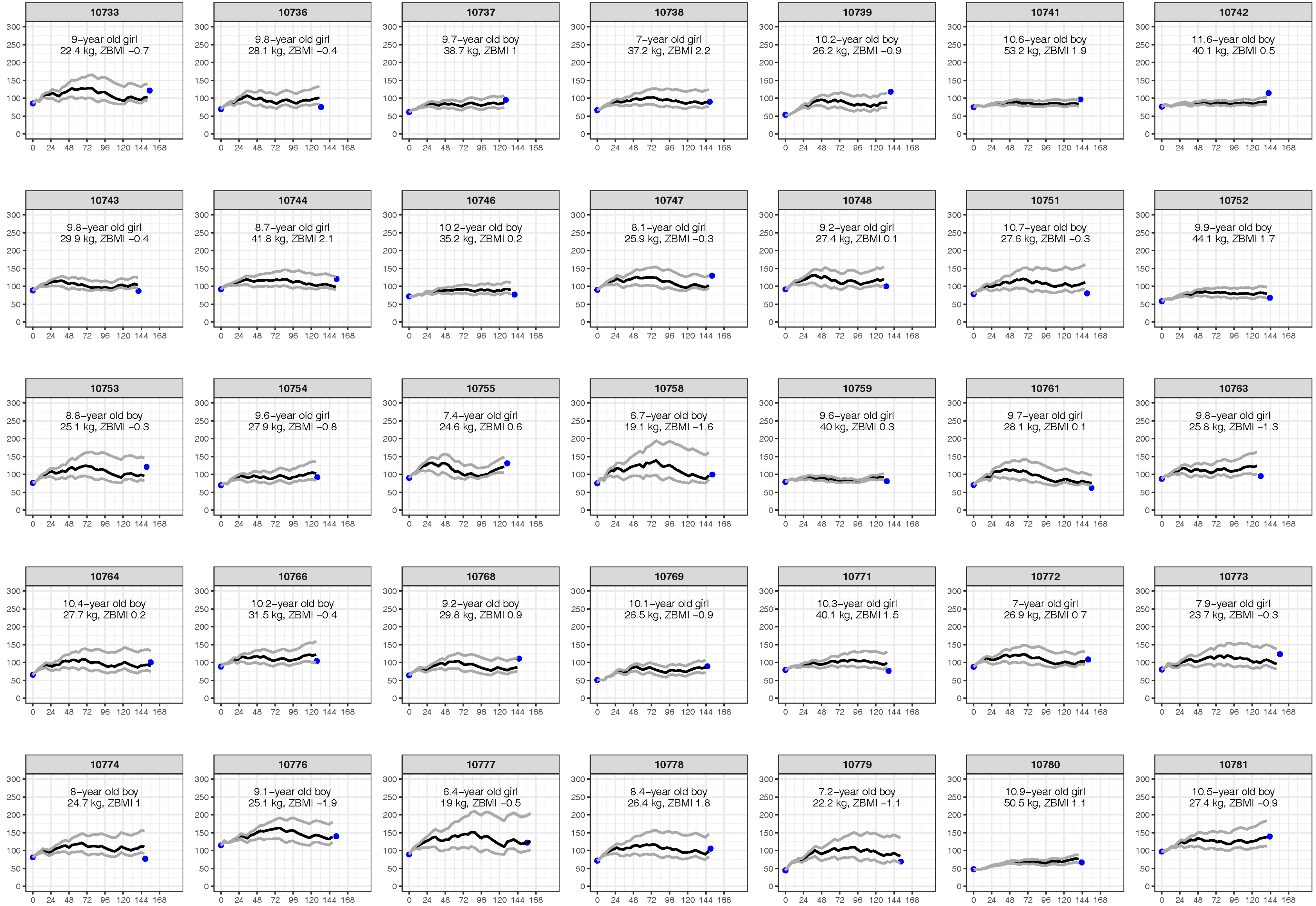
10733
9–year old girl
22.4 kg, ZBMI −0.7
10736
9.8–year old girl
28.1 kg, ZBMI −0.4
10737
9.7–year old boy
38.7 kg, ZBMI 1
10738
7–year old girl
37.2 kg, ZBMI 2.2
10739
10.2–year old boy
26.2 kg, ZBMI −0.9
10741
10.6–year old boy
53.2 kg, ZBMI 1.9
10742
11.6–year old boy
40.1 kg, ZBMI 0.5
10743
9.8–year old girl
29.9 kg, ZBMI −0.4
10744
8.7–year old girl
41.8 kg, ZBMI 2.1
10746
10.2–year old boy
35.2 kg, ZBMI 0.2
10747
8.1–year old girl
25.9 kg, ZBMI −0.3
10748
9.2–year old girl
27.4 kg, ZBMI 0.1
10751
10.7–year old boy
27.6 kg, ZBMI −0.3
10752
9.9–year old boy
44.1 kg, ZBMI 1.7
10753
8.8–year old boy
25.1 kg, ZBMI −0.3
10754
9.6–year old girl
27.9 kg, ZBMI −0.8
10755
7.4–year old girl
24.6 kg, ZBMI 0.6
10758
6.7–year old boy
19.1 kg, ZBMI −1.6
10759
9.6–year old girl
40 kg, ZBMI 0.3
10761
9.7–year old girl
28.1 kg, ZBMI 0.1
10763
9.8–year old girl
25.8 kg, ZBMI −1.3
10764
10.4–year old boy
27.7 kg, ZBMI 0.2
10766
10.2–year old boy
31.5 kg, ZBMI −0.4
10768
9.2–year old boy
29.8 kg, ZBMI 0.9
10769
10.1–year old girl
26.5 kg, ZBMI −0.9
10771
10.3–year old girl
40.1 kg, ZBMI 1.5
10772
7–year old girl
26.9 kg, ZBMI 0.7
10773
7.9–year old girl
23.7 kg, ZBMI −0.3
10774
8–year old boy
24.7 kg, ZBMI 1
10776
9.1–year old boy
25.1 kg, ZBMI −1.9
10777
6.4–year old girl
19 kg, ZBMI −0.5
10778
8.4–year old boy
26.4 kg, ZBMI 1.8
10779
7.2–year old boy
22.2 kg, ZBMI −1.1
10780
10.9–year old girl
50.5 kg, ZBMI 1.1
10781
10.5–year old boy
27.4 kg, ZBMI −0.9

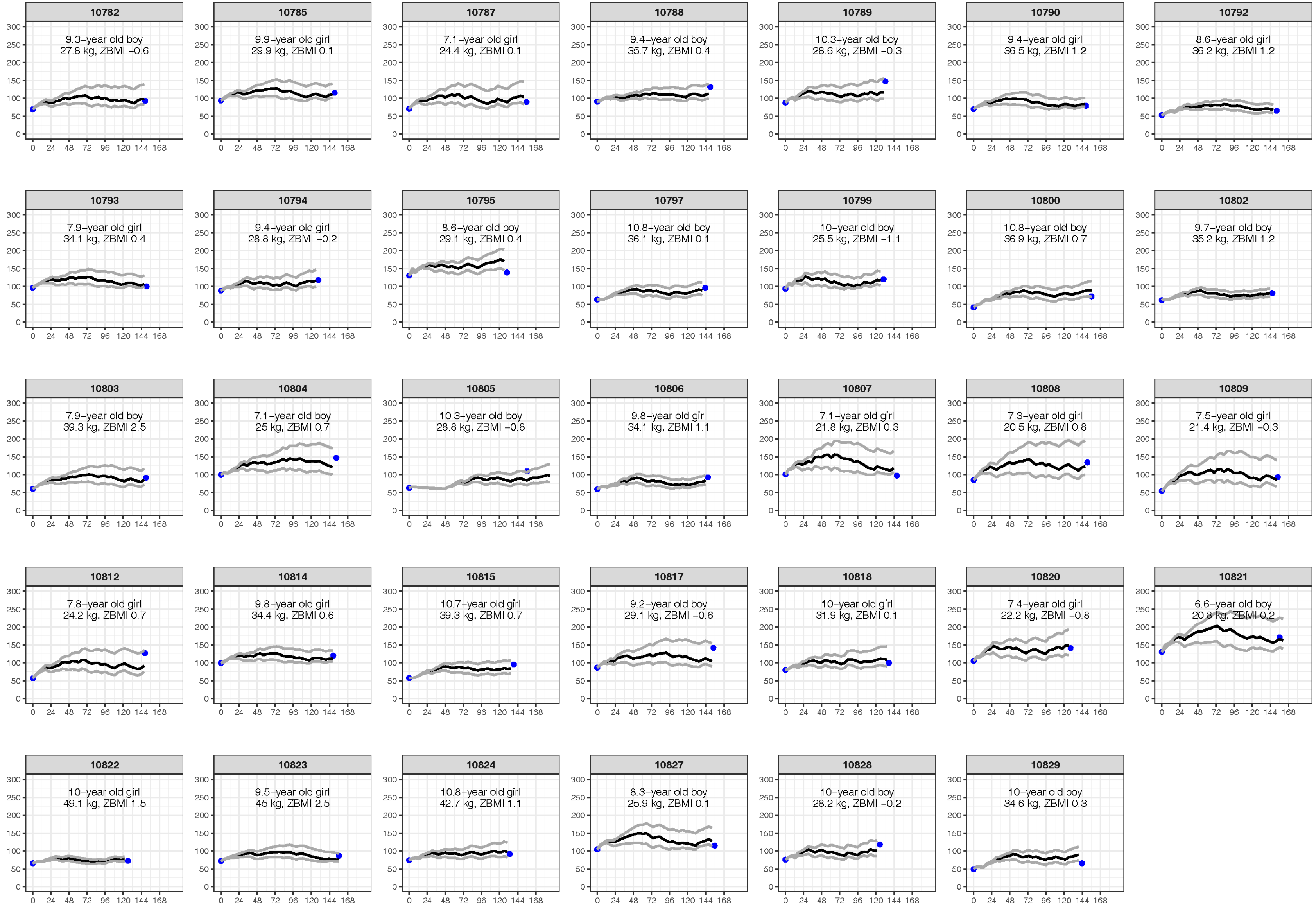
10782
9.3–year old boy
27.8 kg, ZBMI −0.6
10785
9.9–year old girl
29.9 kg, ZBMI 0.1
10787
7.1–year old girl
24.4 kg, ZBMI 0.1
10788
9.4–year old boy
35.7 kg, ZBMI 0.4
10789
10.3–year old boy
28.6 kg, ZBMI −0.3
10790
9.4–year old girl
36.5 kg, ZBMI 1.2
10792
8.6–year old girl
36.2 kg, ZBMI 1.2
10793
7.9–year old girl
34.1 kg, ZBMI 0.4
10794
9.4–year old girl
28.8 kg, ZBMI −0.2
10795
8.6–year old boy
29.1 kg, ZBMI 0.4
10797
10.8–year old boy
36.1 kg, ZBMI 0.1
10799
10–year old boy
25.5 kg, ZBMI −1.1
10800
10.8–year old boy
36.9 kg, ZBMI 0.7
10802
9.7–year old boy
35.2 kg, ZBMI 1.2
10803
7.9–year old boy
39.3 kg, ZBMI 2.5
10804
7.1–year old boy
25 kg, ZBMI 0.7
10805
10.3–year old boy
28.8 kg, ZBMI −0.8
10806
9.8–year old girl
34.1 kg, ZBMI 1.1
10807
7.1–year old girl
21.8 kg, ZBMI 0.3
10808
7.3–year old girl
20.5 kg, ZBMI 0.8
10809
7.5–year old girl
21.4 kg, ZBMI −0.3
10812
7.8–year old girl
24.2 kg, ZBMI 0.7
10814
9.8–year old girl
34.4 kg, ZBMI 0.6
10815
10.7–year old girl
39.3 kg, ZBMI 0.7
10817
9.2–year old boy
29.1 kg, ZBMI −0.6
10818
10–year old girl
31.9 kg, ZBMI 0.1
10820
7.4–year old girl
22.2 kg, ZBMI −0.8
10821
6.6–year old boy
20.8 kg, ZBMI 0.2
10822
10–year old girl
49.1 kg, ZBMI 1.5
10823
9.5–year old girl
45 kg, ZBMI 2.5
10824
10.8–year old girl
42.7 kg, ZBMI 1.1
10827
8.3–year old boy
25.9 kg, ZBMI 0.1
10828
10–year old boy
28.2 kg, ZBMI −0.2
10829
10–year old boy
34.6 kg, ZBMI 0.3
0 50 100 150 200 250 300
0 24 48 72 96 120 144 168

Figure S10. The distribution of residual error at 3 years: observation – population prediction at the 50th percentile. Red vertical line: 50th percentile = 5.4 nmol/L. Dashed vertical lines: 5th and 95th percentiles (-26.2 and 39.5 nmol/L, respectively).

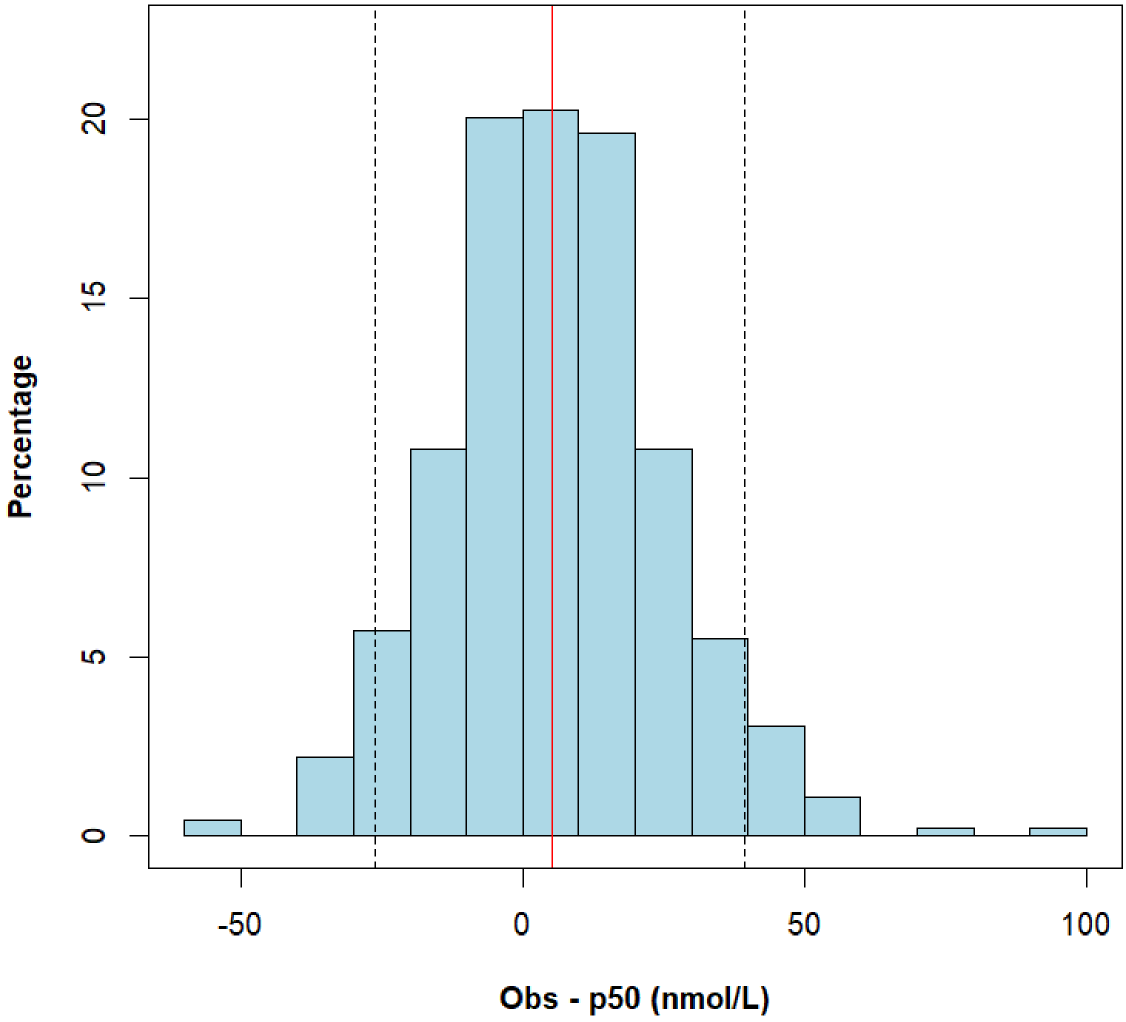

Figure S11. The distribution of the range (i.e. 97.5th – 2.5th percentiles) at 1, 2, and 3 years. Red vertical line: 50th percentile of the range. Dashed vertical lines: 5th and 95th percentiles of the range.

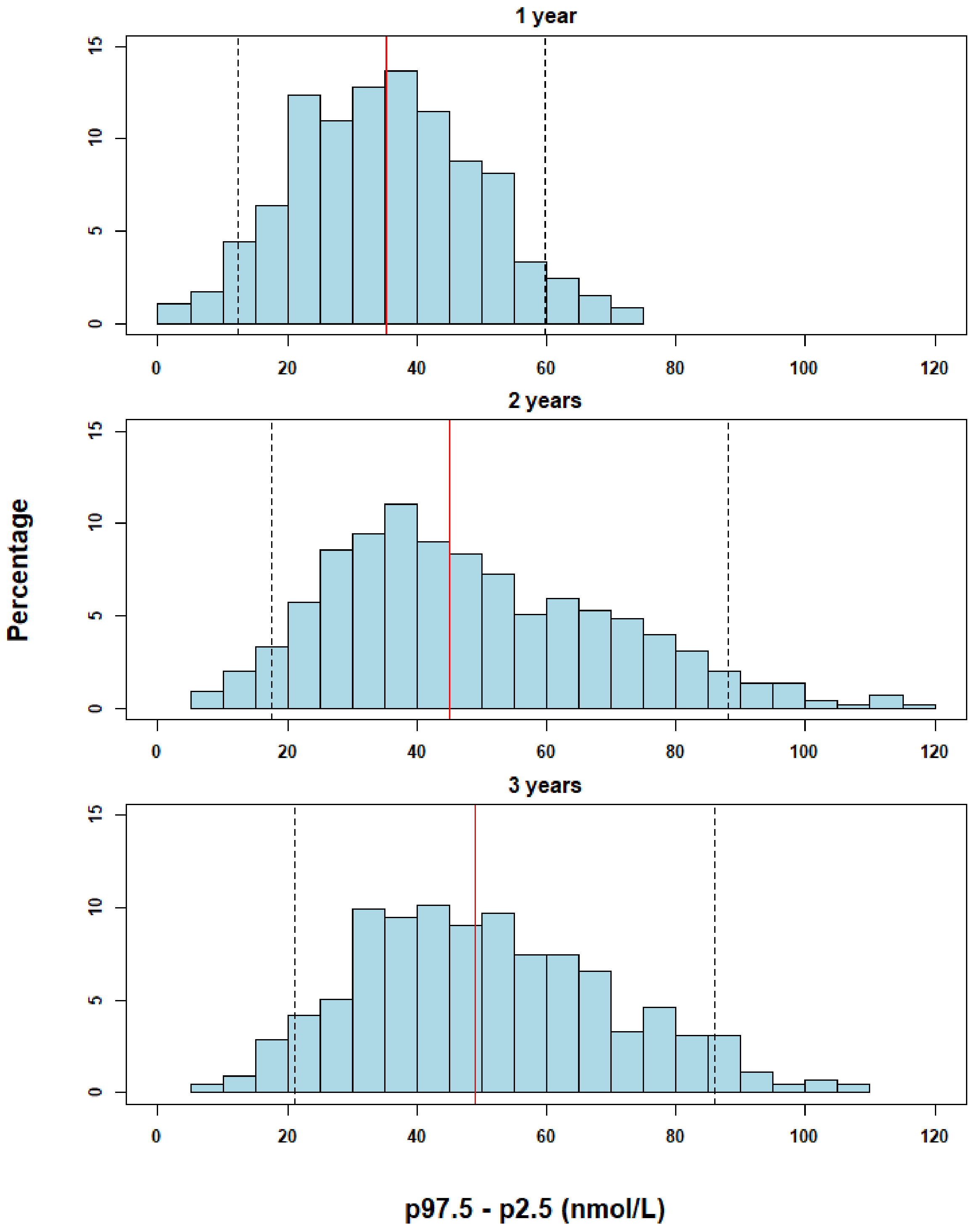

Figure S12. The scatter plots between the 2.5th – 97.5th percentiles range and baseline bodyweight and ZBMI.

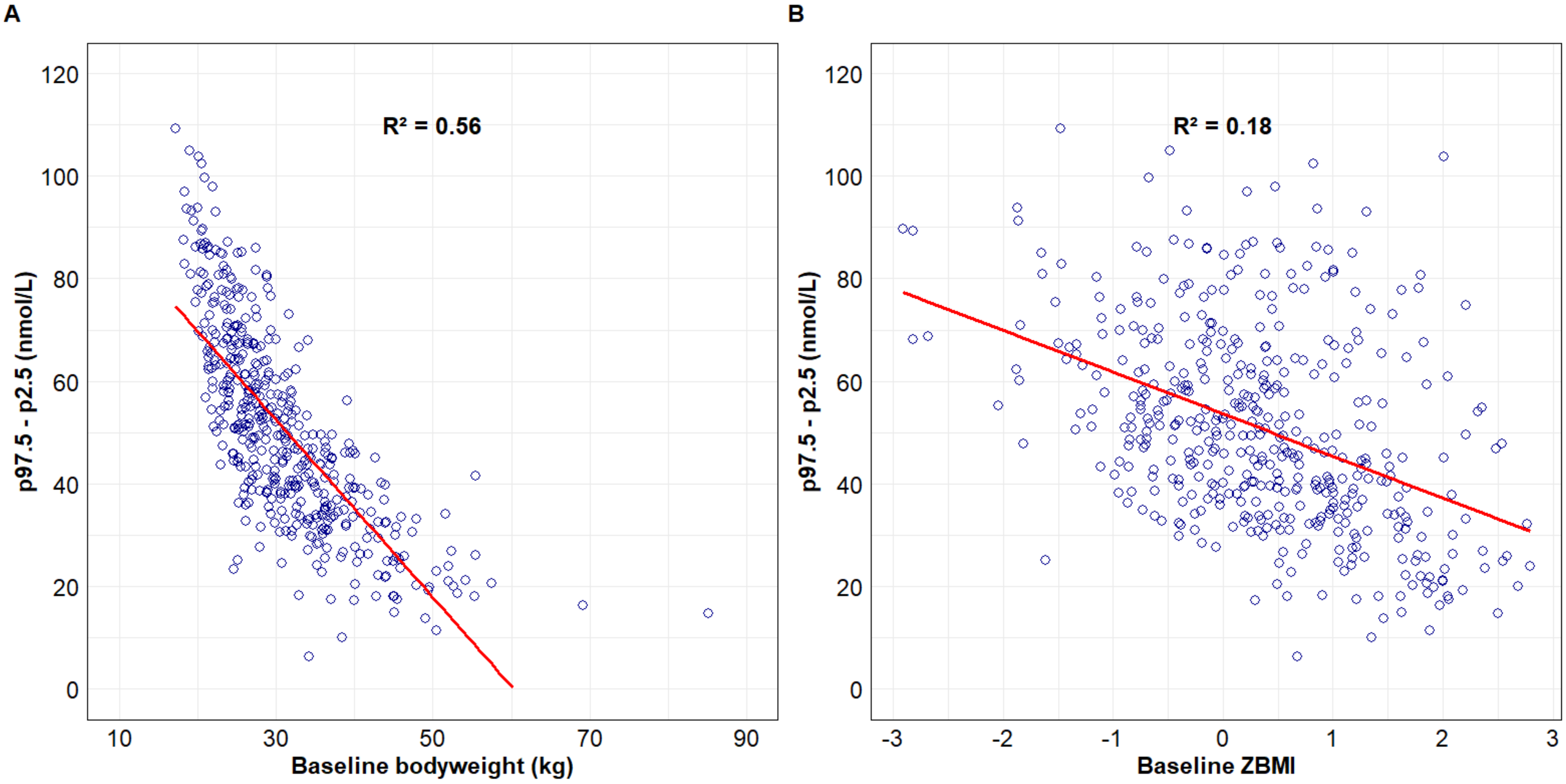

Figure S13. Comparison of the treated Mongolian children (A-E: n = 1756) with treated Cape Town children used to fit the model (F-J: n = 77). For the Mongolian trial, we selected those children (less than 12-year-old) with both baseline and 3-year serum 25(OH)D, gated at the limit of quantification (14.2 nmol/L). Compliance is defined as the ratio between the total number of received doses (Cape Town: 250µg; Mongolia: 350µg) and the number of weeks. It is normalised to 100%. Dashed vertical lines: mean. Salmon: baseline. Cyan: final. For the Mongolian trial, weight was only measured at baseline.

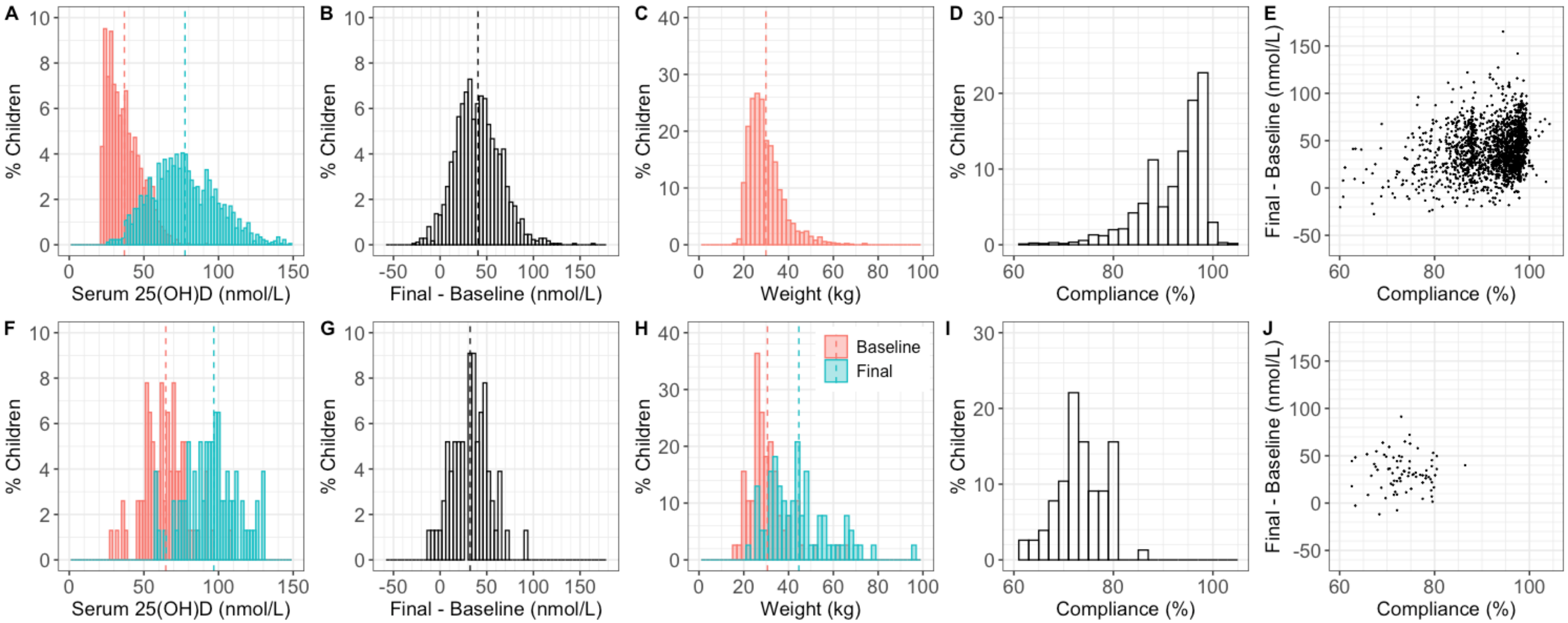

Figure S14. Simulation of serum 25(OH)D concentrations in Mongolian children receiving weekly dose of 350 µg vitamin D_3. Simulated participants were under 12 years at baseline and had both baseline and follow up 25(OH)D_3 above the lower limit of quantification: 1756 children in total (Figures S1 and S13): A) the Cape Town model with typical values: CL_{max} = 0.012 h^{-1}, C_{50} = 86.3 nmol/L; B) CL_{max} = 0.048 h^{-1}; C) C_{50} = 30 nmol/L; D) C_{50} = 10 nmol/L; E) CL_{max} = 0.048 h^{-1}; C_{50} = 30 nmol/L; F) CL_{max} = 0.048 h^{-1}; C_{50} = 110 nmol/L. f: girls; m: boys.

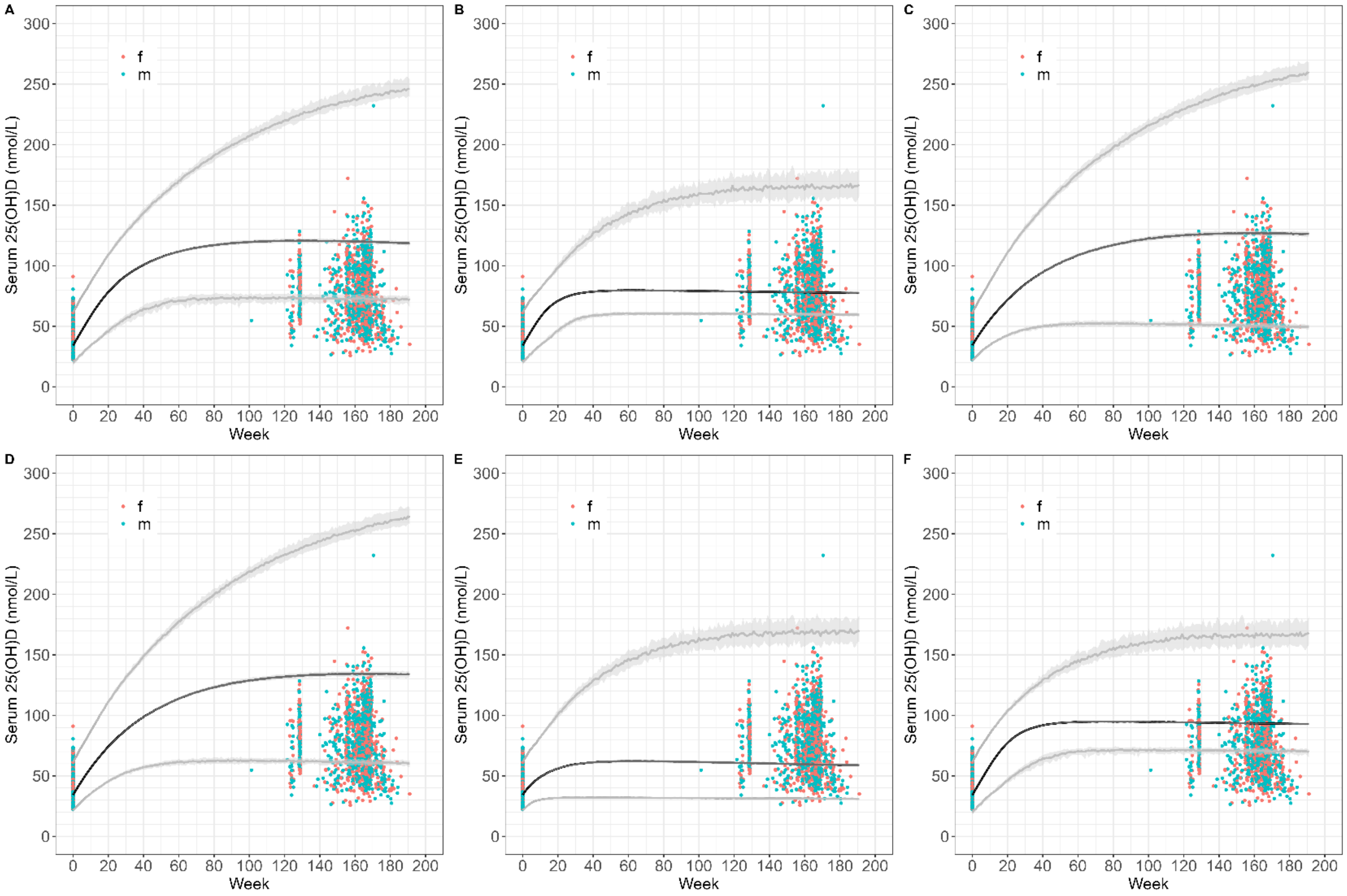

Figure S15. Serum 25(OH)D in the control group of the ViDiKids safety study (n=105) changes with time. A sinusoidal curve was fitted to the data. Amplitude: 11.5 nmol/L.

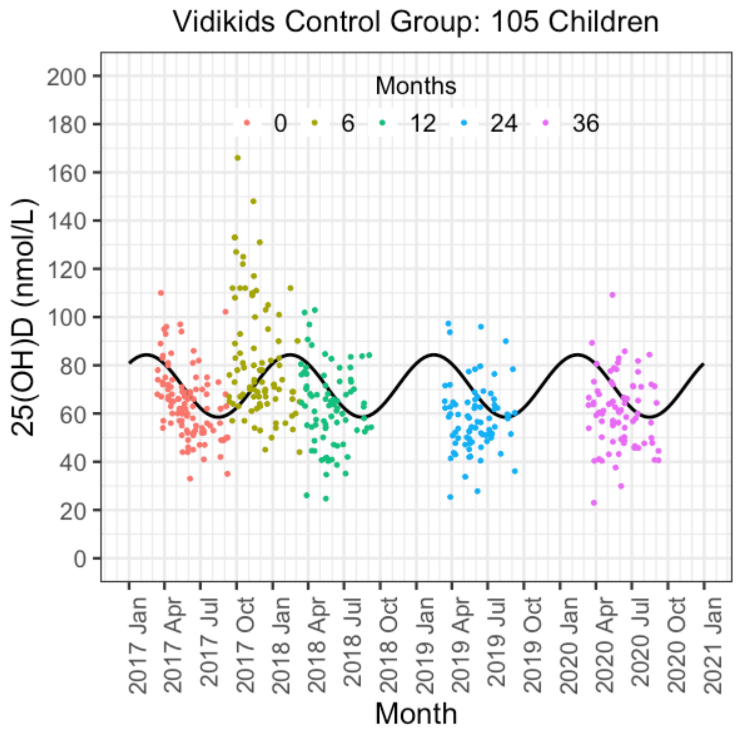

Figure S16. Simulation of serum 25(OH)D concentration at 3 years with weekly 250μg vitamin D_3 dosing at varying values of CL_{max} and C_{50}. A) $CL_{max} = 0.012h^{-1}$, $C_{50} \in [10,200]\ nmol/L$; B) $CL_{max} = 0.01248$, $C_{50} \in [10,200]\ nmol/L$; C) $C_{50} = 30, 86.3, 110\ nmol/L$, $CL_{max} \in [0.001,0.06]h^{-1}$. All plots in semi-log scale. Red dashed line marks the typical value for a Cape Town child. At baseline, weight = 30kg; 25(OH)D = 50 nmol/L. Weight increases by 3.16 kg/year.

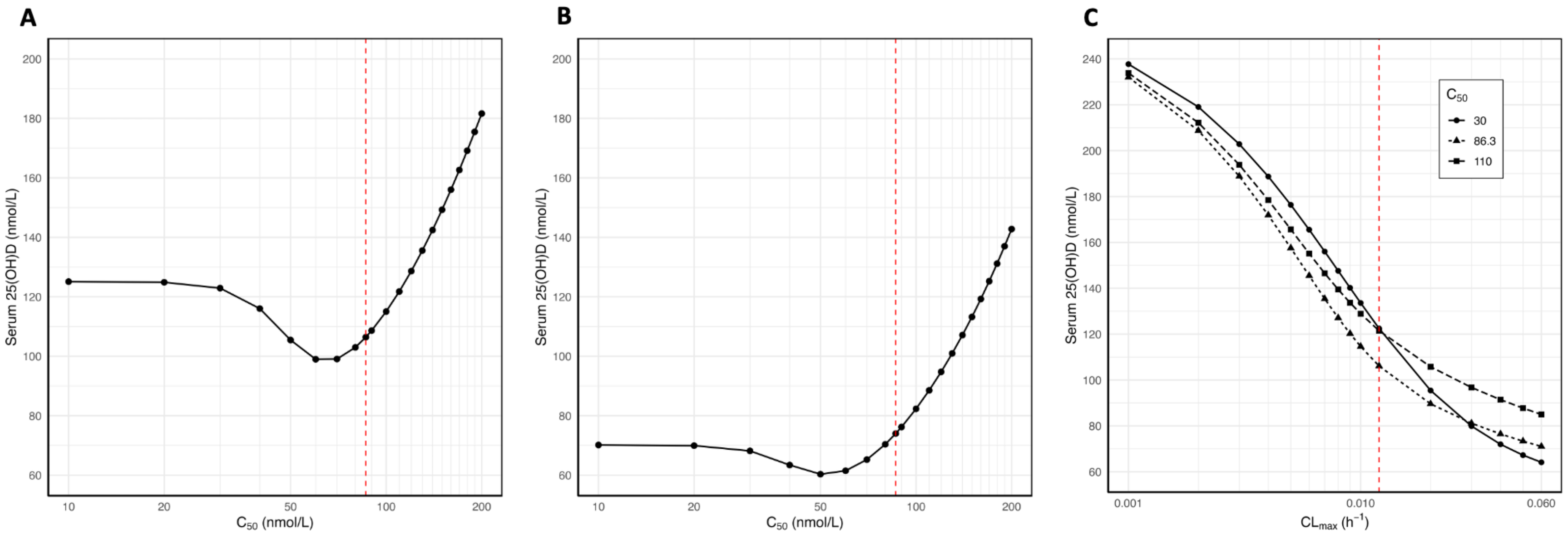

Figure S17. 25(OH)D clearance rate $\frac{CL_{max} \times C_{25D}^{\gamma}}{C_{50}^{\gamma} + C_{25D}^{\gamma}} \times C_{25D}$ vs 25(OH)D. Salmon: healthy adults [14]. Cyan: Cape Town children (mean and 95% CI of CL_{max}: Table 2). Black dashed line: CL_{max} = 0.048 h^{-1}; C_{50} = 30 nmol/L. For healthy adults (salmon) and Cape Town children (cyan): 1000 samples were generated using Latin Hypercube method for CL_{max}, C_{50} and γ; line: expected mean; band: 5% – 95% confidence interval.

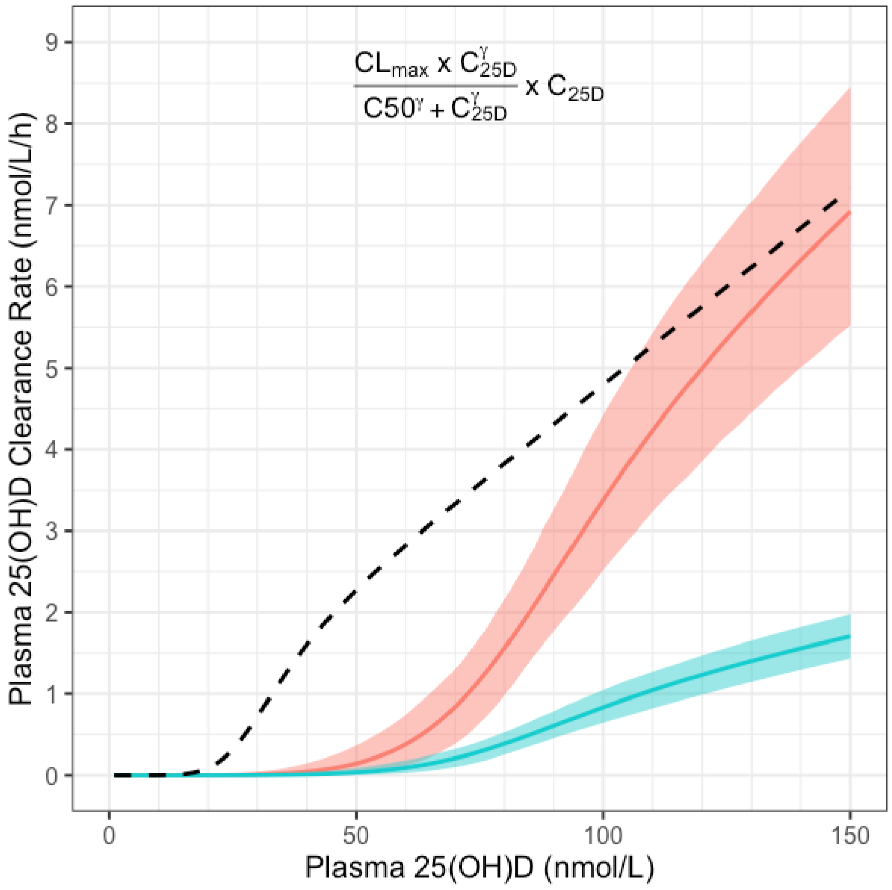

Supplementary Tables

Table S1. Baseline characteristics of the study population.

	**Participants (*n* = 77)**
**Age, y (Mean±SD)**	8.94±1.29
**Male, n (%)**	30(38.96)
**Weight, kg (Mean±SD)**	30.57±8.99
**Height, cm (Mean±SD)**	149±10.91
**BMI, kg/m^2 (Mean±SD)**	17.75±3.46
**ZBMI, n (%)**	
**-3 to -2**	1 (1.30)
**-2 to -1**	7 (9.09)
**-1 to +1**	49 (63.64)
**+1 to +2**	14 (18.18)
**+2 to +3**	6 (7.79)
**25(OH)D, nmol/L (Mean±SD)**	64.67±14.85

Data are presented as number with percentage in parenthesis or as mean and standard deviation.

Abbreviation: BMI, body mass index; ZBMI, BMI for age z-score.

Table S2. Physiological parameters used for paediatric compartmental lumping.

Compartment	Blood flow (%) *	Blood flow (L/h) #	Volume (%)	Volume (L) §	Source
**Lung**	100	273	0.76	0.23	Brown1997 and Edginton 2006
**Heart**	4	10.92	0.47	0.14	Brown1997
**Kidney**	17.5	47.78	0.44	0.13	Brown1997
**Liver, total**	22.7	61.97	2.57	0.77	Brown1997
**Liver, hepatic artery**	4.6	12.56	-	-	Brown1997
**GI**	15.1	41.22	1.71	0.51	adjusted
**Spleen**	3	8.19	0.26	0.08	adjusted
**Brain**	11.4	31.12	2	0.60	Brown1997
**Adipose**	5.2	14.20	21.42	6.43	Brown1997
**Skin**	5.8	15.83	3.71	1.11	Brown1997
**Muscle**	19.1	52.14	40	12	Brown1997
**Bone**	4.2	11.47	14.29	4.29	Brown1997
**Venous**	100	273	6	1.80	Brown1997
**Arterial**	100	273	2	0.60	Brown1997
**Splanchnic organ**	18.1	49.413	na		Brown1997
**Rest of the body (all other organs)**	10.1	27.57	4.37	1.31	Brown1997

* Blood flow (%) comes from (Brown *et al*, 1997)

# Edginton (2006) reported mean cardiac output for boys (4.8 L/min) and girls (4.3 L/min) aged between 7 and 9 years. We take the average of 4.55 L/min (273 L/h). Subsequently, blood flow to each organ was calculated by multiplying the first column (blood flow %) with the total mean cardiac output.

§ We assumed an apparent density of 1 kg/L. For a 30 kg bodyweight, total volume is 30L.

Brown RP, Delp MD, Lindstedt SL, Rhomberg LR and Beliles RP (1997) Physiological parameter values for physiologically based pharmacokinetic models. *Toxicology and industrial health* **13**:407-484.

Edginton AN, Schmitt W and Willmann S (2006) Development and Evaluation of a Generic Physiologically Based Pharmacokinetic Model for Children. *Clinical Pharmacokinetics* **45**:1013-1034.

Table S3. Physiological parameters of the PBPK model for paediatrics after compartments are lumped together.

Parameters	Definition	Units	Value
**Qco**	Cardiac output	L/h	273.00
**Ql**	Blood flow to liver	L/h	61.97
**Qrb**	Blood flow to rest of body	L/h	211.03
**Vven**	Volume of venous compartment	L	1.80
**Vl**	Volume of liver compartment	L	0.77
**Vrb**	Volume of rest of body compartment	L	26.83
**Vart**	Volume of arterial compartment	L	0.6

Note: Lumping: Assume apparent density = 1kg/L. Vrb = 30L – Vl – Vven – Vart; Qrb = Qco – Ql.

Table S4. Drug-specific parameters for model development.

Parameter	Description	Unit	Value
**Vitamin D**			
K_a	Absorption rate	h^{-1}	0.19
Kp_l	Partition coefficient for liver	Dimensionless	1
Kp_{rb}	Partition coefficient for rest of the body	Dimensionless	0.09
MCLH	Hepatic clearance after repeated dose	h^{-1}	0.222
**25(OH)D**			
$Kp25_l$	Partition coefficient for liver	Dimensionless	1
C_{50}	Concentration at half maximum clearance	nmol/L	86.3
γ	Exponent	Dimensionless	5.64

Table S5. Model development for 25(OH)D paediatric pharmacokinetics.

	Fixed Effects	Random Effects	Covariate	AIC	BIC	OBJFV	Comments
1	CL_{MAX}<br>$Kp25_{rb}$	$CL_{max}=e^{TCL_{max}+\eta_{CL_{max}}}$<br>$K_{p25rb}=e^{TK_{p25rb}+\eta_{K_{p25rb}}}$<br>$cor\left(\eta_{CL_{max}}, \eta_{K_{p25rb}}\right) = 0.204$	-	2408	2434	1846	Base model
2	CL_{MAX}<br>$Kp25_{rb}$	$CL_{max}=e^{TCL_{max}+\eta_{CL_{max}}}$<br>$K_{p25rb}=e^{TK_{p25rb}+\eta_{K_{p25rb}}}$<br>$cor\left(\eta_{CL_{max}}, \eta_{K_{p25rb}}\right) = -0.668$	$V_{art} = TV_{art} \times \frac{WT}{30}$<br>$V_{ven} = TV_{ven} \times \frac{WT}{30}$<br>$V_{rb} = TV_{rb} \times \frac{WT}{30}$<br>$V_{l} = TV_{l} \times \frac{WT}{30}$	2400	2426	1838	Baseline weight is used to predict the volume of distribution.<br>The objective function value is improved compared to model 1.
3	CL_{MAX}<br>$Kp25_{rb}$	$CL_{max}=e^{TCL_{max}+\eta_{CL_{max}}} \times \left(\frac{WT}{30}\right)^{0.75}$<br>$K_{p25rb}=e^{TK_{p25rb}+\eta_{K_{p25rb}}}$<br>$cor\left(\eta_{CL_{max}}, \eta_{K_{p25rb}}\right) = -0.754$	$CL_{max}=e^{TCL_{max}+\eta_{CL_{max}}} \times \left(\frac{WT}{30}\right)^{0.75}$	2388	2413	1826	Baseline weight is used to predict clearance.<br>The objective function value is improved compared to model 2.
4	CL_{MAX}<br>$Kp25_{rb}$	$CL_{max}=e^{TCL_{max}+\eta_{CL_{max}}} \times \left(\frac{WT_{cur}}{30}\right)^{0.75}$<br>$K_{p25rb}=e^{TK_{p25rb}+\eta_{K_{p25rb}}}$<br>$cor\left(\eta_{CL_{max}}, \eta_{K_{p25rb}}\right) = -0.781$	$CL_{max}=e^{TCL_{max}+\eta_{CL_{max}}} \times \left(\frac{WT}{30}\right)^{0.75}$<br>$V_{art} = TV_{art} \times \frac{WT}{30}$<br>$V_{ven} = TV_{ven} \times \frac{WT}{30}$<br>$V_{rb} = TV_{rb} \times \frac{WT}{30}$<br>$V_{l} = TV_{l} \times \frac{WT}{30}$	2387	2413	1825	Baseline weight is used to predict the volume of distribution and clearance.<br>The objective function value is not significantly different from model 3.
**5**	CL_{MAX}<br>$Kp25_{rb}$	$CL_{max}=e^{TCL_{max}+\eta_{CL_{max}}} \times \left(\frac{WT_{cur}}{30}\right)^{0.75}$<br>$K_{p25rb}=e^{TK_{p25rb}+\eta_{K_{p25rb}}}$<br>$cor\left(\eta_{CL_{max}}, \eta_{K_{p25rb}}\right) = -0.731$	$WT_{cur} = WT + 3.0836 \times \left(\frac{time}{24\times365\times3}\right)$ for boy<br>$WT_{cur} = WT + 3.2282 \times \left(\frac{time}{24\times365\times3}\right)$ for girl	2527	2552	1965	A changing weight is used to predict the volume of distribution and clearance in a sex-dependent manner.

	Fixed Effects	Random Effects	Covariate	AIC	BIC	OBJFV	Comments
			$CL_{max}=e^{TCL_{max}+\eta_{CL_{max}}}\times(\frac{WT_{cur}}{30})^{0.75}$<br>$K_{p25rb}=e^{TK_{p25rb}+\eta_{K_{p25rb}}}$<br>$V_{art} = TV_{art} \times \frac{WT_{cur}}{30}$<br>$V_{ven} = TV_{ven} \times \frac{WT_{cur}}{30}$<br>$V_{rb} = TV_{rb} \times \frac{WT_{cur}}{30}$<br>$V_{l} = TV_{l} \times \frac{WT_{cur}}{30}$				The objective function value is higher than model 4.
**6**	CL_{MAX}<br>$Kp25_{rb}$	$CL_{max}=e^{TCL_{max}+\eta_{CL_{max}}}\times(\frac{WT_{cur}}{30})^{0.75}$<br>$K_{p25rb}=e^{TK_{p25rb}+\eta_{K_{p25rb}}+(ZBMI\times K_{p25rb_{ZBMI}})}$<br>$cor\left(\eta_{CL_{max}}, \eta_{K_{p25rb}}\right)$=-0.758	$WT_{cur} = WT + 3.0836 \times (\frac{time}{24\times365\times3})$ for boy<br>$WT_{cur} = WT + 3.2282 \times (\frac{time}{24\times365\times3})$ for girl<br>$CL_{max}=e^{TCL_{max}+\eta_{CL_{max}}}\times(\frac{WT_{cur}}{30})^{0.75}$<br>$K_{p25rb}=e^{TK_{p25rb}+\eta_{K_{p25rb}}+(ZBMI\times K_{p25rb_{ZBMI}})}$<br>$V_{art} = TV_{art} \times \frac{WT_{cur}}{30}$<br>$V_{ven} = TV_{ven} \times \frac{WT_{cur}}{30}$<br>$V_{rb} = TV_{rb} \times \frac{WT_{cur}}{30}$<br>$V_{l} = TV_{l} \times \frac{WT_{cur}}{30}$	2535	2564	1971	A changing weight is used to predict the volume of distribution and clearance in a sex-dependent manner. ZBMI is used as a covariate for the partition coefficient of the rest of the body.<br>The objective function value is higher than 4.
**7**	CL_{MAX}<br>$Kp25_{rb}$	$CL_{max}=e^{TCL_{max}+\eta_{CL_{max}}}\times(\frac{WT_{cur}}{30})^{0.75}$<br>$K_{p25rb}=e^{TK_{p25rb}+\eta_{K_{p25rb}}}$<br>$cor\left(\eta_{CL_{max}}, \eta_{K_{p25rb}}\right)$ = -0.760	$WT_{cur} = WT + (WT_{3Y} - WT) \times \frac{time}{24\times365\times3}$<br>$CL_{max}=e^{TCL_{max}+\eta_{CL_{max}}}\times(\frac{WT_{cur}}{30})^{0.75}$	2401	2426	1839	A changing weight is used to predict the volume of distribution and clearance. Weight is interpolated

	Fixed Effects	Random Effects	Covariate	AIC	BIC	OBJFV	Comments
			$V_{art} = TV_{art} \times \frac{WT_{cur}}{30}$<br>$V_{ven} = TV_{ven} \times \frac{WT_{cur}}{30}$<br>$V_{rb} = TV_{rb} \times \frac{WT_{cur}}{30}$<br>$V_{l} = TV_{l} \times \frac{WT_{cur}}{30}$				linearly between the baseline and final time points.<br>The objective function value is higher than model 4.
**8**	CL_{MAX}<br>$Kp25_{rb}$	$CL_{max}=e^{TCL_{max}+\eta_{CL_{max}}} \times (\frac{WT_{cur}}{30})^{0.75}$<br>$K_{p25rb}=e^{TK_{p25rb}+\eta_{K_{p25rb}}}$<br>$cor\left(\eta_{CL_{max}}, \eta_{K_{p25rb}}\right) = -0.204$	$WT_{cur} = WT + (WT_{3Y} - WT) \times \frac{time}{24\times365\times3}$<br>$CL_{max}=e^{TCL_{max}+\eta_{CL_{max}}} \times (\frac{WT_{cur}}{30})^{0.75}$<br>$V_{art} = TV_{art} \times \frac{WT_{cur}}{30}$<br>$V_{ven} = TV_{ven} \times \frac{WT_{cur}}{30}$<br>$V_{rb} = TV_{rb} \times \frac{WT_{cur}}{30}$<br>$V_{l} = TV_{l} \times \frac{WT_{cur}}{30}$<br>$cp=\frac{A25_{ven}}{V_{ven}}-1.9$; time= 24×365<br>$cp=\frac{A25_{ven}}{V_{ven}}-6.6$; time= 24×365×2<br>$cp=\frac{A25_{ven}}{V_{ven}}-4.1$; time= 24×365×3	3078	3104	2516	A changing weight is used to predict the volume of distribution and clearance. Weight is interpolated linearly between the baseline and final time points.<br>Plasma concentration is calculated at a specific time point (“cp”) subtracting the mean value of the control group at each time point.<br>The objective function value is higher than model 4.
**9a**	CL_{MAX}<br>$Kp25_{fm}$<br>($Kp25_{lm}$ = 1)	$CL_{max}=e^{TCL_{max}+\eta_{CL_{max}}} \times (\frac{WT_{cur}}{30})^{0.75}$<br>$K_{p25fm}=e^{TKp25_{fm}}$	$WT_{cur} = WT + (WT_{3Y} - WT) \times \frac{time}{24\times365\times3}$<br>$CL_{max}=e^{TCL_{max}+\eta_{CL_{max}}} \times (\frac{WT_{cur}}{30})^{0.75}$	2385	2404	1828	A changing weight is used to predict the volume of distribution and clearance. Weight is interpolated

	Fixed Effects	Random Effects	Covariate	AIC	BIC	OBJFV	Comments
			$V_{art} = TV_{art} \times \frac{WT_{cur}}{30}$<br>$V_{ven} = TV_{ven} \times \frac{WT_{cur}}{30}$<br>$V_{rb} = TV_{rb} \times \frac{WT_{cur}}{30}$<br>$V_{l} = TV_{l} \times \frac{WT_{cur}}{30}$<br>$V_{fm} = FM_0+(FM_{3Y}-FM_0)\times\frac{time}{24\times365\times3}$<br>$V_{lm} = LM_0+(lM_{3Y}-LM_0)\times\frac{time}{24\times365\times3}$				linearly between the baseline and final time points.<br>ZBMI is used to predict fat mass and lean mass.*<br>The objective function value is marginally higher than model 4, while the AIC and BIC are improved.
**9b**	CL_{MAX}<br>$Kp25_{fm}$<br>($Kp25_{lm}$ = 2)	$CL_{max}=e^{TCL_{max}+\eta_{CL_{max}}} \times(\frac{WT_{cur}}{30})^{0.75}$<br>$K_{p25fm}=e^{TKp25_{fm}}$	$WT_{cur} = WT + (WT_{3Y} - WT) \times \frac{time}{24\times365\times3}$<br>$CL_{max}=e^{TCL_{max}+\eta_{CL_{max}}} \times(\frac{WT_{cur}}{30})^{0.75}$<br>$V_{art} = TV_{art} \times \frac{WT_{cur}}{30}$<br>$V_{ven} = TV_{ven} \times \frac{WT_{cur}}{30}$<br>$V_{rb} = TV_{rb} \times \frac{WT_{cur}}{30}$<br>$V_{l} = TV_{l} \times \frac{WT_{cur}}{30}$<br>$V_{fm} = FM_0+(FM_{3Y}-FM_0)\times\frac{time}{24\times365\times3}$<br>$V_{lm} = LM_0+(lM_{3Y}-LM_0)\times\frac{time}{24\times365\times3}$	2371	2389	1813	A changing weight is used to predict the volume of distribution and clearance. Weight is interpolated linearly between the baseline and final time points.<br>ZBMI is used to predict fat mass and lean mass.*<br>The objective function value, AIC and BIC are improved compared to model 9a.
**9c**	CL_{MAX}<br>$Kp25_{fm}$<br>($Kp25_{lm}$ = 3)	$CL_{max}=e^{TCL_{max}+\eta_{CL_{max}}} \times(\frac{WT_{cur}}{30})^{0.75}$<br>$K_{p25fm}=e^{TKp25_{fm}}$	$WT_{cur} = WT + (WT_{3Y} - WT) \times \frac{time}{24\times365\times3}$<br>$CL_{max}=e^{TCL_{max}+\eta_{CL_{max}}} \times(\frac{WT_{cur}}{30})^{0.75}$	2360	2379	1802	A changing weight is used to predict the volume of distribution and clearance. Weight is interpolated

	Fixed Effects	Random Effects	Covariate	AIC	BIC	OBJFV	Comments
			$V_{art} = TV_{art} \times \frac{WT_{cur}}{30}$<br>$V_{ven} = TV_{ven} \times \frac{WT_{cur}}{30}$<br>$V_{rb} = TV_{rb} \times \frac{WT_{cur}}{30}$<br>$V_{l} = TV_{l} \times \frac{WT_{cur}}{30}$<br>$V_{fm} = FM_0+(FM_{3Y}-FM_0)\times\frac{time}{24\times365\times3}$<br>$V_{lm} = LM_0+(lM_{3Y}-LM_0)\times\frac{time}{24\times365\times3}$				linearly between the baseline and final time points.<br>ZBMI is used to predict fat mass and lean mass.*<br>The objective function value, AIC and BIC are improved compared to model 9b.
**9d**	CL_{MAX}<br>$Kp25_{fm}$<br>($Kp25_{lm}$ = 4)	$CL_{max}=e^{TCL_{max}+\eta_{CL_{max}}}\times(\frac{WT_{cur}}{30})^{0.75}$<br>$K_{p25fm}=e^{TKp25_{fm}}$	$WT_{cur} = WT + (WT_{3Y} - WT) \times \frac{time}{24\times365\times3}$<br>$CL_{max}=e^{TCL_{max}+\eta_{CL_{max}}}\times(\frac{WT_{cur}}{30})^{0.75}$<br>$V_{art} = TV_{art} \times \frac{WT_{cur}}{30}$<br>$V_{ven} = TV_{ven} \times \frac{WT_{cur}}{30}$<br>$V_{rb} = TV_{rb} \times \frac{WT_{cur}}{30}$<br>$V_{l} = TV_{l} \times \frac{WT_{cur}}{30}$<br>$V_{fm} = FM_0+(FM_{3Y}-FM_0)\times\frac{time}{24\times365\times3}$<br>$V_{lm} = LM_0+(lM_{3Y}-LM_0)\times\frac{time}{24\times365\times3}$	2353	2372	1795	A changing weight is used to predict the volume of distribution and clearance. Weight is interpolated linearly between the baseline and final time points.<br>ZBMI is used to predict fat mass and lean mass.*<br>The objective function value, AIC and BIC are improved compared to model 9c.<br>**Best model**
**9e**	CL_{MAX}<br>$Kp25_{fm}$<br>($Kp25_{lm}$ = 6)	$CL_{max}=e^{TCL_{max}+\eta_{CL_{max}}}\times(\frac{WT_{cur}}{30})^{0.75}$<br>$K_{p25fm}=e^{TKp25_{fm}}$	$WT_{cur} = WT + (WT_{3Y} - WT) \times \frac{time}{24\times365\times3}$<br>$CL_{max}=e^{TCL_{max}+\eta_{CL_{max}}}\times(\frac{WT_{cur}}{30})^{0.75}$	2353	2371	1795	A changing weight is used to predict the volume of distribution and clearance. Weight is interpolated

	Fixed Effects	Random Effects	Covariate	AIC	BIC	OBJFV	Comments
			$V_{art} = TV_{art} \times \frac{WT_{cur}}{30}$<br>$V_{ven} = TV_{ven} \times \frac{WT_{cur}}{30}$<br>$V_{rb} = TV_{rb} \times \frac{WT_{cur}}{30}$<br>$V_{l} = TV_{l} \times \frac{WT_{cur}}{30}$<br>$V_{fm} = FM_0+(FM_{3Y}-FM_0)\times\frac{time}{24\times365\times3}$<br>$V_{lm} = LM_0+(lM_{3Y}-LM_0)\times\frac{time}{24\times365\times3}$				linearly between the baseline and final time points.<br>ZBMI is used to predict fat mass and lean mass.*<br>The objective function value, AIC and BIC are similar to model 9d.
**9f**	CL_{MAX}<br>$Kp25_{fm}$<br>($Kp25_{lm}$ = 8)	$CL_{max}=e^{TCL_{max}+\eta_{CL_{max}}}\times(\frac{WT_{cur}}{30})^{0.75}$<br>$K_{p25fm}=e^{TKp25_{fm}}$	$WT_{cur} = WT + (WT_{3Y} - WT) \times \frac{time}{24\times365\times3}$<br>$CL_{max}=e^{TCL_{max}+\eta_{CL_{max}}}\times(\frac{WT_{cur}}{30})^{0.75}$<br>$V_{art} = TV_{art} \times \frac{WT_{cur}}{30}$<br>$V_{ven} = TV_{ven} \times \frac{WT_{cur}}{30}$<br>$V_{rb} = TV_{rb} \times \frac{WT_{cur}}{30}$<br>$V_{l} = TV_{l} \times \frac{WT_{cur}}{30}$<br>$V_{fm} = FM_0+(FM_{3Y}-FM_0)\times\frac{time}{24\times365\times3}$<br>$V_{lm} = LM_0+(lM_{3Y}-LM_0)\times\frac{time}{24\times365\times3}$	2378	2397	1821	A changing weight is used to predict the volume of distribution and clearance. Weight is interpolated linearly between the baseline and final time points.<br>ZBMI is used to predict fat mass and lean mass.*<br>The objective function value, AIC and BIC are worse compared to model 9d.
**10**	CL_{MAX}<br>$Kp25_{fm}$<br>$Kp25_{lm}$	$CL_{max}=e^{TCL_{max}+\eta_{CL_{max}}}\times(\frac{WT_{cur}}{30})^{0.75}$<br>$K_{p25fm}=e^{TKp25_{fm}}$	$WT_{cur} = WT + (WT_{3Y} - WT) \times \frac{time}{24\times365\times3}$<br>$CL_{max}=e^{TCL_{max}+\eta_{CL_{max}}}\times(\frac{WT_{cur}}{30})^{0.75}$	2409	2435	1847	A changing weight is used to predict the volume of distribution and clearance. Weight is interpolated

	Fixed Effects	Random Effects	Covariate	AIC	BIC	OBJFV	Comments
		$K_{p25lm}=e^{TKp25_{lm}}$	$V_{art} = TV_{art} \times \frac{WT_{cur}}{30}$<br>$V_{ven} = TV_{ven} \times \frac{WT_{cur}}{30}$<br>$V_{rb} = TV_{rb} \times \frac{WT_{cur}}{30}$<br>$V_{l} = TV_{l} \times \frac{WT_{cur}}{30}$<br>$V_{fm} = FM_0+(FM_{3Y}-FM_0)\times\frac{time}{24\times365\times3}$<br>$V_{lm} = LM_0+(lM_{3Y}-LM_0)\times\frac{time}{24\times365\times3}$				linearly between the baseline and final time points.<br>ZBMI is used to predict fat mass and lean mass.<br>One more fixed and random effects than Model 9: $Kp25l_m$.<br>The objective function value, AIC and BIC are worse compared to model 9d.
**11**	CL_{MAX}<br>$Kp25_{rb}$<br>CL_{C50}	$CL_{max}=e^{TCL_{max}+\eta_{CL_{max}}}\times(\frac{WT}{30})^{0.75}$<br>$K_{p25rb}=e^{TK_{p25rb}+\eta_{K_{p25rb}}}$<br><br>$cor\left(\eta_{CL_{max}}, \eta_{K_{p25rb}}\right) = -0.266$	$CL_{max}=e^{TCL_{max}+\eta_{CL_{max}}}\times(\frac{WT}{30})^{0.75}$	2372	2401	1808	Weight is used to predict clearance.<br>The objective function value, AIC and BIC are worse compared to model 9d.

*Fat mass and lean mass are calculated from ZBMI using the following equations:

$$f_{FM}=\begin{cases}\max(\frac{28.61+7.82\times ZBMI-0.91\times ZBMI^2+0.03\times ZBMI^3}{100}, 0.05), if\ ZBMI \le 6.19\\ 0.49, f\ ZBMI > 6.19\end{cases}$$

$FM_0 = WT\times f_{FM}(0)$

$FM_{3Y} = WT_{3Y}\times f_{fm}(3Y)$

$LM_0 = WT\times(1-f_{FM}(0)) - (1.80 + 0.77 + 0.6)\times\frac{WT}{30}$

$LM_{3Y} = WT\times(1-f_{FM}(3Y)) - (1.80 + 0.77 + 0.6)\times\frac{WT}{30}$

Table S6. 25(OH)D model fitting results.

25(OH)D Model 1: “fit.s1.25OHD.rds”

	Est.	SE	%RSE	Back-transformed(95%CI)	IIV(CV%)	Shrink(SD)%
TCL_EMAX	-4.01	0.0736	1.83	0.0181 (0.0156, 0.0209)	63.4	6.54%<
TKp25rb	1.51	0.0919	6.07	4.54 (3.8, 5.44)	32.7	34.3%>
C50	FIXED	FIXED	FIXED	86.3		
γ	FIXED	FIXED	FIXED	5.64		
add.err	0.00249			0.00249		
prop.err	0.106			0.106		

25(OH)D Model 2: “fit.s2.25OHD.rds”

	Est.	SE	%RSE	Back-transformed(95%CI)	IIV(CV%)	Shrink(SD)%
TCL_EMAX	-4.01	0.0794	1.98	0.0181 (0.0155, 0.0212)	70.3	2.05%<
TKp25rb	1.4	0.0869	6.2	4.06 (3.43, 4.82)	37.0	25.6%=
C50	FIXED	FIXED	FIXED	86.3		
γ	FIXED	FIXED	FIXED	5.64		
add.err	0.00249			0.00249		
prop.err	0.104			0.104		

25(OH)D Model 3: “fit.s3.25OHD.rds”

	Est.	SE	%RSE	Back-transformed(95%CI)	IIV(CV%)	Shrink(SD)%
TCL_EMAX	-4	0.0779	1.95	0.0184 (0.0158, 0.0214)	68.3	5.93%<
TKp25rb	1.15	0.114	9.93	3.16 (2.52, 3.95)	69.7	9.53%<
C50	FIXED	FIXED	FIXED	86.3		
γ	FIXED	FIXED	FIXED	5.64		
add.err	0.00252			0.00252		
prop.err	0.102			0.102		

25(OH)D Model 4: “fit.s4.25OHD.rds”

	Est.	SE	%RSE	Back-transformed(95%CI)	IIV(CV%)	Shrink(SD)%
TCL_EMAX	-3.99	0.0715	1.79	0.0186 (0.0161, 0.0214)	60.6	1.69%<
TKp25rb	1.32	0.0857	6.47	3.76 (3.18, 4.45)	34.1	10.5%<
C50	FIXED	FIXED	FIXED	86.3		
γ	FIXED	FIXED	FIXED	5.64		
add.err	0.00249			0.00249		
prop.err	0.106			0.106		

25(OH)D Model 5: “fit.s5.25OHD.rds”

	Est.	SE	%RSE	Back-transformed(95%CI)	IIV(CV%)	Shrink(SD)%
TCL_EMAX	-3.99	0.0759	1.9	0.0185 (0.0159, 0.0215)	66.4	7.64%<
TKp25rb	1.28	0.0999	7.77	3.61 (2.97, 4.39)	53.6	11.9%<
C50	FIXED	FIXED	FIXED	86.3		
γ	FIXED	FIXED	FIXED	5.64		
add.err	0.0025			0.0025		
prop.err	0.102			0.102		

25(OH)D Model 6: “fit.s6.25OHD.rds”

	Est.	SE	%RSE	Back-transformed(95%CI)	IIV(CV%)	Shrink(SD)%
TCL_EMAX	-3.99	0.0741	1.86	0.0185 (0.016, 0.0214)	63.7	3.21%<
TKp25rb	1.35	0.0941	6.94	3.88 (3.22, 4.66)	36.2	16.2%<
Kp25rb_ZBMI	0.0137	0.073	533	0.0137 (-0.129, 0.157)		
C50	FIXED	FIXED	FIXED	86.3		
γ	FIXED	FIXED	FIXED	5.64		
add.err	0.0025			0.0025		
prop.err	0.104			0.104		

25(OH)D Model 7: “fit.s7.25OHD.rds”

	Est.	SE	%RSE	Back-transformed(95%CI)	IIV(CV%)	Shrink(SD)%
TCL_EMAX	-4.59	0.0785	1.71	0.0102 (0.00873, 0.0119)	64.3	17.8%<
TKp25rb	1.2	0.124	10.3	3.33 (2.61, 4.25)	113.	15.9%<
C50	FIXED	FIXED	FIXED	86.3		
γ	FIXED	FIXED	FIXED	5.64		
add.err	0.00247			0.00247		
prop.err	0.0936			0.0936		

25(OH)D Model 8: “fit.s8.25OHD.rds”

	Est.	SE	%RSE	Back-transformed(95%CI)	IIV(CV%)	Shrink(SD)%
TCL_EMAX	-4.37	0.107	2.44	0.0127 (0.0103, 0.0156)	9.79	38.9%>
TKp25rb	0.944	0.296	31.4	2.57 (1.44, 4.59)	48.6	45.8%>
C50	FIXED	FIXED	FIXED	86.3		
γ	FIXED	FIXED	FIXED	5.64		
add.err	37.8			37.8		
prop.err	0.000353			0.000353		

25(OH)D Model 9a: “fit.s9a.25OHD.rds”

	Est.	SE	%RSE	Back-transformed(95%CI)	IIV(CV%)	Shrink(SD)%
TCL_EMAX	-4.42	0.0795	1.8	0.012 (0.0103, 0.014)	61.6	9.01%<
TKp25fm	2.34	0.0632	2.7	10.4 (9.16, 11.7)		
C50	FIXED	FIXED	FIXED	86.3		
γ	FIXED	FIXED	FIXED	5.64		
Kp25lm	FIXED	FIXED	FIXED	1		
add.err	0.00251			0.00251		
prop.err	0.111			0.111		

25(OH)D Model 9b: “fit.s9b.25OHD.rds”

	Est.	SE	%RSE	Back-transformed(95%CI)	IIV(CV%)	Shrink(SD)%
TCL_EMAX	-4.43	0.0781	1.76	0.0119 (0.0102, 0.0139)	61.0	9.85%<
TKp25fm	2.13	0.0771	3.62	8.4 (7.22, 9.77)		
C50	FIXED	FIXED	FIXED	86.3		
γ	FIXED	FIXED	FIXED	5.64		
Kp25lm	FIXED	FIXED	FIXED	2		
add.err	0.00265			0.00265		
prop.err	0.11			0.11		

25(OH)D Model 9c: “fit.s9c.25OHD.rds”

	Est.	SE	%RSE	Back-transformed(95%CI)	IIV(CV%)	Shrink(SD)%
TCL_EMAX	-4.41	0.0778	1.76	0.0121 (0.0104, 0.0141)	61.8	10.9%<
TKp25fm	1.81	0.104	5.74	6.14 (5, 7.53)		
C50	FIXED	FIXED	FIXED	86.3		
γ	FIXED	FIXED	FIXED	5.64		
Kp25lm	FIXED	FIXED	FIXED	3		
add.err	0.00249			0.00249		
prop.err	0.11			0.11		

25(OH)D Model 9d: “fit.s9d.25OHD.rds”

	Est.	SE	%RSE	Back-transformed(95%CI)	IIV(CV%)	Shrink(SD)%
TCL_EMAX	-4.43	0.0786	1.77	0.0119 (0.0102, 0.0139)	62.7	13.1%<
TKp25fm	1.54	0.138	8.96	4.66 (3.56, 6.11)		
C50	FIXED	FIXED	FIXED	86.3		
γ	FIXED	FIXED	FIXED	5.64		
Kp25lm	FIXED	FIXED	FIXED	4		
add.err	0.00282			0.00282		
prop.err	0.109			0.109		

25(OH)D Model 9e: “fit.s9e.25OHD.rds”

	Est.	SE	%RSE	Back-transformed(95%CI)	IIV(CV%)	Shrink(SD)%
TCL_EMAX	-4.49	0.0814	1.81	0.0112 (0.00957, 0.0132)	65.3	13.7%<
TKp25fm	0.694	0.311	44.8	2 (1.09, 3.68)		
C50	FIXED	FIXED	FIXED	86.3		
γ	FIXED	FIXED	FIXED	5.64		
Kp25lm	FIXED	FIXED	FIXED	6		
add.err	0.00251			0.00251		
prop.err	0.11			0.11		

25(OH)D Model 9f: “fit.s9f.25OHD.rds”

	Est.	SE	%RSE	Back-transformed(95%CI)	IIV(CV%)	Shrink(SD)%
TCL_EMAX	-4.65	0.0909	1.96	0.0096 (0.00803, 0.0115)	74.9	16.8%<
TKp25fm	0.121	0.204	169	1.13 (0.756, 1.68)		
C50	FIXED	FIXED	FIXED	86.3		
γ	FIXED	FIXED	FIXED	5.64		
Kp25lm	FIXED	FIXED	FIXED	8		
add.err	0.00265			0.00265		
prop.err	0.119			0.119		

25(OH)D Model 10: “fit.s9a.25OHD.rds”

	Est.	SE	%RSE	Back-transformed(95%CI)	IIV(CV%)	Shrink(SD)%
TCL_EMAX	-4.46	0.0779	1.75	0.0116 (0.00996, 0.0135)	59.3	7.77%<
TKp25lm	1.36	0.104	7.68	3.89 (3.17, 4.77)		
TKp25fm	1.6	0.118	7.34	4.96 (3.94, 6.25)		
C50	FIXED	FIXED	FIXED	86.3		
γ	FIXED	FIXED	FIXED	5.64		
add.err	0.00245			0.00245		
prop.err	0.107			0.107		
eta.FM_frac					1.36	39.8%>

25(OH)D Model 11: “fit.s9a.25OHD.rds”

	Est.	SE	%RSE	Back-transformed(95%CI)	IIV(CV%)	Shrink(SD)%
TCL_EMAX	-3.24	0.32	9.89	0.0392 (0.021, 0.0735)	105.	1.11%<
TKp25rb	1.37	0.119	8.69	3.95 (3.12, 4.98)	44.1	37.4%>
C50	4.76	0.0841	1.77	117 (98.8, 137)		
γ	FIXED	FIXED	FIXED	5.64		
add.err	0.00252			0.00252		
prop.err	0.0995			0.0995		
